# Compaction and Segregation of DNA in *Escherichia coli*

**DOI:** 10.3390/life14060660

**Published:** 2024-05-22

**Authors:** Conrad L. Woldringh

**Affiliations:** Faculty of Science, Swammerdam Institute for Life Sciences (SILS), University of Amsterdam, 1098 XH Amsterdam, The Netherlands; c.woldringh@icloud.com

**Keywords:** bacterial nucleoid, phase-contrast microscopy, DNA polymer physics, protein depletion, chromosome arms, replication bubble, orisome, active DNA segregation, passive DNA segregation

## Abstract

Theoretical and experimental approaches have been applied to study the polymer physics underlying the compaction of DNA in the bacterial nucleoid. Knowledge of the compaction mechanism is necessary to obtain a mechanistic understanding of the segregation process of replicating chromosome arms (replichores) during the cell cycle. The first part of this review discusses light microscope observations demonstrating that the nucleoid has a lower refractive index and thus, a lower density than the cytoplasm. A polymer physics explanation for this phenomenon was given by a theory discussed at length in this review. By assuming a phase separation between the nucleoid and the cytoplasm and by imposing equal osmotic pressure and chemical potential between the two phases, a minimal energy situation is obtained, in which soluble proteins are depleted from the nucleoid, thus explaining its lower density. This theory is compared to recent views on DNA compaction that are based on the exclusion of polyribosomes from the nucleoid or on the transcriptional activity of the cell. These new views prompt the question of whether they can still explain the lower refractive index or density of the nucleoid. In the second part of this review, we discuss the question of how DNA segregation occurs in *Escherichia coli* in the absence of the so-called active ParABS system, which is present in the majority of bacteria. How is the entanglement of nascent chromosome arms generated at the origin in the parental DNA network of the *E. coli* nucleoid prevented? Microscopic observations of the position of fluorescently-labeled genetic loci have indicated that the four nascent chromosome arms synthesized in the initial replication bubble segregate to opposite halves of the sister nucleoids. This implies that extensive intermingling of daughter strands does not occur. Based on the hypothesis that leading and lagging replichores synthesized in the replication bubble fold into microdomains that do not intermingle, a passive four-excluding-arms model for segregation is proposed. This model suggests that the key for segregation already exists in the structure of the replication bubble at the very start of DNA replication; it explains the different patterns of chromosome arms as well as the segregation distances between replicated loci, as experimentally observed.

## 1. Introduction

In contrast to the chromatin in eukaryotic cells, bacterial DNA occurs freely in cells and is not surrounded by a membrane. Nevertheless, this DNA can be observed as a distinct central region, called the nucleoid. What macromolecular interactions or activities induce this phase separation and cause the compaction of DNA? 

As early as 1956, Mason and Powelson [1] showed, by phase-contrast microscopy of cells grown in a rich medium supplemented with gelatin, that a bright central structure expands and divides in step with the growing cell. They concluded that they were observing “bacterial nuclei”. At that time, it was still assumed that bacteria contained “nuclei” or nuclear structures and that they possessed “a mitotic apparatus consisting of a centriole and spindle” (for a discussion of the changing views on the bacterial nucleoid, see Robinow and Kellenberger [2]). 

The light appearance of the nucleoid structure indicates its low refractive index (RI) and thus, its low concentration or density of macromolecules. Why is the nucleoid’s DNA not dispersed throughout the whole cell, and how do the distinct low-density regions, as observed by phase-contrast microscopy, originate? These questions have been studied by many groups by applying molecular dynamics simulations [3,4], which will not be discussed here. These questions have also been addressed in theoretical studies based on equilibrium statistical mechanics and on formulations of the free energy of cell systems. In those studies [5,6], the interactions between supercoiled DNA and macromolecular crowders such as soluble proteins and polyribosomes were defined, allowing for the calculation of the volume of the nucleoid.

In his depletion theory [5], Odijk developed equations for the osmotic pressure and chemical potential of the nucleoid and cytoplasm, assuming a phase separation, as observed microscopically. His calculations show that a minimal free-energy situation can be obtained in the cell if soluble proteins are depleted from the nucleoid, in agreement with the observed lower density and RI value of the nucleoid. While Odijk [5] merely considered the interaction of supercoiled DNA with soluble proteins, the Männik group [6,7] also analyzed the crowding interaction of DNA with larger particles, such as polyribosomes. They concluded that the depletion of soluble proteins is not needed to obtain a compact nucleoid; however, they did not consider the nucleoid’s lower density.

In the first part of this review, we compare the old phase-contrast observations of Mason and Powelson to recent microscopic studies that confirm their work (Section 2.1). Because the calculations in Odijk’s depletion theory [5] are difficult to understand, we briefly discuss the basic concepts used by polymer physicists in Section 2.2 (see also [8]). These calculations are summarized step by step in Appendix A. They will have to be reconsidered because of the new fluorescence microscope estimates of cell and nucleoid volumes in living cells, presented in Section 2.3. 

In Section 3.1, we compare the different approaches of Odijk [5] and Männik’s group [6] regarding the question of whether soluble proteins have to be depleted from the nucleoid in order to obtain an RI difference between the nucleoid and the cytoplasm, as observed by phase-contrast microscopy. The relevant data and assumptions to calculate the RI value for the cells of Männik’s group [6] and for new estimates of living cells, presented in Section 2.3, are summarized in Appendix B. 

In Section 3.2, we discuss alternative explanations for nucleoid compaction. They involve the consideration of the solvent quality of the RNA-containing cytoplasm for DNA [9] and transcriptional activities that induce the folding of transcribed and supercoiled regions [10,11]. Also, for these studies, the question that remains to be answered is whether the low refractive index of the nucleoid is ensured. Because the effect of transcription inhibition on nucleoid volume is an important factor in these studies, we reviewed the literature on the microscopy of rifampicin-treated *E. coli* cells.

In the second part of this review, the physical mechanism(s) of polymers involved in DNA compaction are also assumed to influence the process of DNA segregation. Because replication and segregation go hand in hand (see below), the movement of replicated DNA strands through the parental non-replicated DNA network starts at initiation within the initial replication bubble or orisome [12]. From here, the newly synthesized chromosome arms, which contain leading and lagging strands, have to segregate to separate the halves of the daughter nucleoids, as described by Sherratt’s [13] and Hansen’s [14] groups. There are two views, not mutually exclusive, that try to explain the disentanglement of replicated chromosome arms and the formation of daughter nucleoids. The first view is based on an active segregation mechanism such as the tripartite ParAB-*parS* system [15], present in the majority of bacterial species [16,17,18]. This system may be helped by the structural maintenance of chromosome (SMC) proteins and loop extrusion mechanisms [19,20]. The second view considers a passive segregation process based on de novo DNA synthesis. Here, we propose that newly synthesized chromosome arms do not become mixed or entangled because of their physical differences (explained in Section 4.1). From initiation onward, the four nascent arms are maintained as separate entities, which exclude each other while replicating and forming (micro)domains that enlarge and become rearranged in the long axis of the cell. This behavior is proposed in the four-excluding-arms model presented in Section 4.2. The model explains not only how the two daughter arms with their replichores segregate into the two halves of the newly formed nucleoids in the prospective daughter cells [13,14], but also how the different segregation patterns for replicated loci are obtained. Calculations of the volumes of the domains at different stages of replication and the segregation distances between replicated loci pairs are summarized in Appendix C. 

## 2. The Nucleoid as a Low-Density Structure

The phase-contrast microscopy observations of Mason and Powelson [1] demonstrate in a simple way that the bacterial nucleoid represents a low-density region in the cell. The explanation for this, however, is complex and involves microscopic and polymer physics aspects. Some of these will be discussed in this section.

### 2.1. Phase-Contrast Microscopy and Immersive Refractometry 

**Figure 1:**
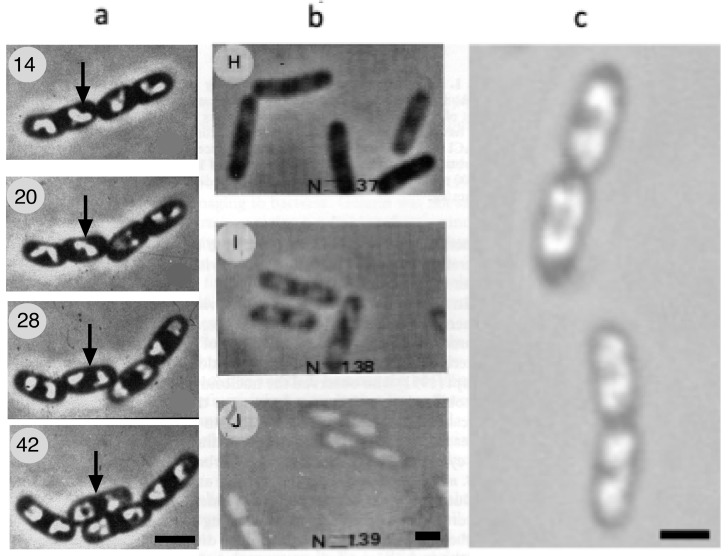
Visualization of bacterial nucleoid in live *E. coli* cells from three laboratories, applying the immersion of cells in medium with a high refractive index. The nucleoids become visible when the refractive indices of the cytoplasm and immersion medium are almost equal in the range of *n* = 1.38–1.39. (**a**) Mason and Powelson [1] used 20% gelatin for the immersion medium and a phase-contrast microscope that showed dark areas in the cells, comparable to the nuclei of stained preparations (negative phase contrast). Here, the image contrast is inverted. The images represent the Figures 4, 7, 9 and 12 in [1] ©1956 American Society for Microbiology. Modified with permission. The age of cells in minutes is indicated in upper left corner. Black arrows indicate the growth and division of a single nucleoid. Magnification bar = 2 µm. (**b**) Cutout from [22]. Note the increase in refractive index of the medium in panels H (*n* = 1.37), I (*n* = 1.38) and J (*n* = 1.39), achieved with increased concentrations of bovine serum albumin (BSA) and reduced concentrations of salt in the medium to maintain constant osmolality. Panel J shows “phase reversal”: the light intensity of the nucleoid is higher than that of the surrounding medium. Magnification bar = 1 µm. (**c**) Still from a movie by Hironori Niki [24] of rapidly growing *E. coli* cells immersed in gelatin, obtained with permission from Niki (Microbial Physiology Laboratory, Department of Gene Function and Phenomics, National Institute of Genetics, Shizuoka, Japan). Magnification bar = 1 µm.

The observations of Mason and Powelson [1] (Figure 1a) were confirmed by studies using polyvinylpyrrolidone (PVP) [21] or bovine serum albumin (BSA) [22] (Figure 1b) to increase the refractive index of the external medium surrounding the cells. As discussed by Barer et al. [23], an increase in the density of the immersion medium reduces the light scattering of a dense bacterium and abolishes the disturbing halo around the cell. At the same time, the phase contrast of internal structures within the cell, such as the nucleoid, is increased. In addition, this immersion technique enables us to estimate the refractive index and thus, the density of the internal structure when its light intensity is equal to that of an external medium with a known concentration. Mason and Powelson [1] argued that the structures with an observed low refractive index did not arise from an effect of gelatin on the cell because the same structures could be seen after fixation and specific staining of DNA with the Feulgen reaction. 

Mason and Powelson [1] also showed how the bright central structure in the cell expands and divides in step with the growing cell. Likewise, a movie of rapidly growing *E. coli* cells immersed in gelatin, made by Yamaichi and Niki [24], shows how the light nucleoid areas enlarge and divide (Figure 1c), confirming that DNA replication and nucleoid segregation go hand in hand [25].

**Figure 2:**
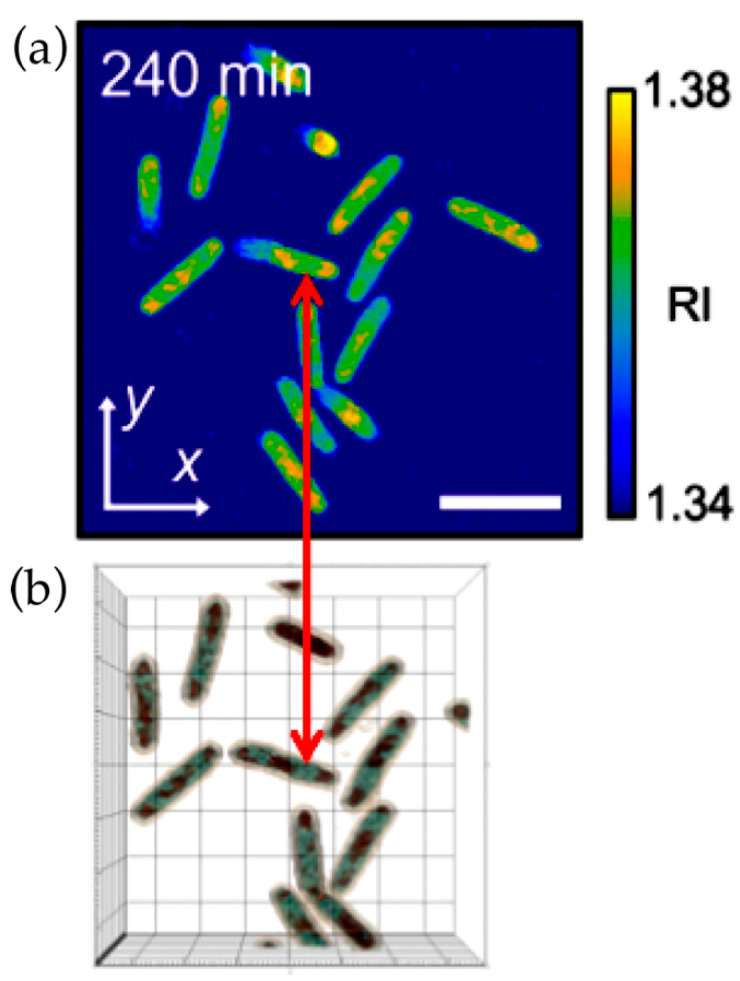
Cutout of Figure 2a,b in Oh et al. [27]. *E. coli* cells were grown in tryptic soy broth in the absence of antibiotics. The doubling time at 25 °C was 56 min. (**a**) A three-section view of the reconstructed refractive index (RI) distribution of *E. coli* cells grown for 240 min without antibiotics. The color map shows the RI values. (**b**) A 3D rendered image of the growing cells in (**a**) obtained with Tomocube software. The double red arrow shows a cell with 2 nucleoids with a lower RI value than cytoplasmic regions. Scale bar = 3 µm. Permission obtained from Yongkeun Park (Department of Physics, Korea Advanced Institute of Science and Technology, Daejeon, Republic of Korea).

Recently, a reduction in density of the nucleoid compared to the cytoplasm was confirmed by spatial light interference microscopy (SLIM). Using this microscopic method, Oldewurtel et al. [26] observed, in *E. coli* cells growing in media without gelatin or BSA, a decrease of about 10–30% in the refractive index at the site of the nucleoid. It is reassuring to know that structures with a low refractive index could also be directly visualized in growing bacteria by optical diffraction tomography (also called digital holographic microscopy) [27] without having to immerse cells in a dense medium (Figure 2). In this study, the nucleoid shows a lower refractive index (1.35) compared to the cytoplasm (1.37) (Figure 2); these values are somewhat lower than those obtained by Valkenburg and Woldringh [28], as discussed below. 

**Figure 3:**
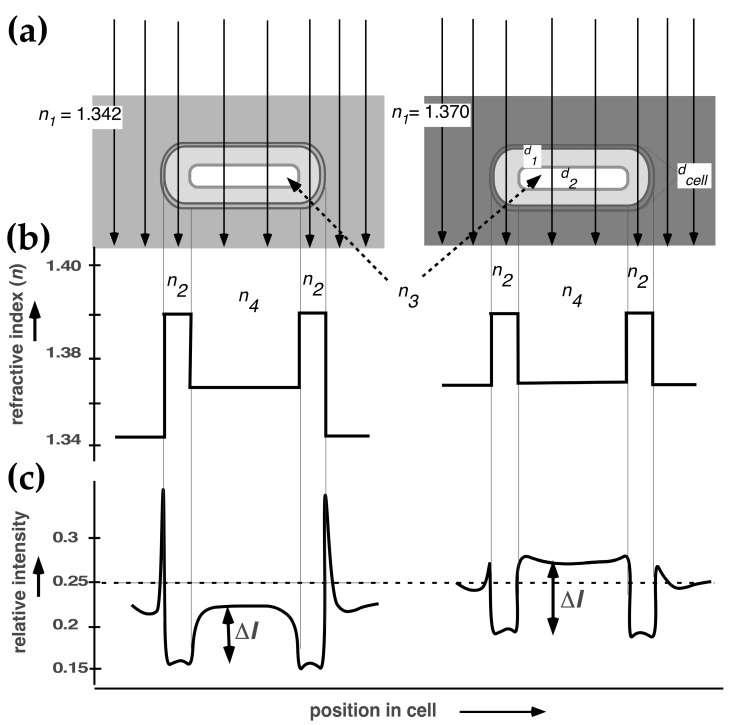
Immersive refractometry of an *E. coli* B/r cell growing in alanine medium with a doubling time of 150 min. (**a**) Thin arrows represent light along the optical axis. For real cell dimensions, see Figure A2 in [28]: cell diameter *d_cell_* = 0.5 µm; thickness of the cytoplasmic layer surrounding the nucleoid *d*_1_ = 0.145 µm; diameter of the nucleoid *d*_2_ = 0.21 µm. In the left and right panels, the cell is immersed in 17.5 and 24% BSA, with refractive index (*n*_1_) values of 1.342 and 1.370, respectively. (**b**) The bold line indicates the refractive index integrated along the optical axis for different cell positions: cytoplasm (*n*_2_), cell center (cytoplasmic layer plus nucleoid, *n*_4_), and nucleoid (dashed arrow, *n*_3_). (**c**) The calculated relative light intensity of the phase-contrast image is indicated as in Figure 5 in [32]. The background intensity is assumed to be equal (horizontal dashed line). Note that the difference in the relative light intensity (Δ*I*) of the nucleoid-containing part (*n*_4_) increases in the medium with a higher refractive index, and the disturbing halo around the cell decreases, improving the visibility of the nucleoid.

Applying immersive refractometry, Valkenburg and Woldringh [28] determined the RI values of the cytoplasm and nucleoid in slow-growing *E. coli* B/r cells (Figure 3). They also calculated theoretical RI values, using the data of Churchward and Bremer [29] for the macromolecular composition of *E. coli* and the measurements of cellular and nucleoid volumes of their cells. For these volume measurements, they used an early confocal scanning light microscope (CSLM) developed by Brakenhoff et al. [30] (for a review of its rediscovery, see Nanninga [31]). This microscope had improved optical resolution and visualized the unstained nucleoid by mere absorption contrast (see Figure 1 in [28]). Assuming that the nucleoid only contained DNA and that all proteins and RNA were located in the cytoplasm, the theoretical RI value for the nucleoid was lower than the experimental value. To match both RI values, it was proposed that the nucleoid must contain, in addition to DNA, about 8.6% proteins, whereas the cytoplasm contains 21% protein and RNA. These values resulted in about 30% reduction in the macromolecular density of the nucleoid compared to the cytoplasm [28]. The next sections discuss the question of whether these observations can be explained based on polymer physics considerations.

### 2.2. Polymer Physics Explanation of Low-Density Nucleoid Based on Odijk’s Depletion Theory

Can the phase separation and reduced density of macromolecules in the nucleoid compared to the cytoplasm described in Figure 1, Figure 2 and Figure 3 be explained by polymer physics interactions between DNA and proteins? It is generally accepted that macromolecular crowding, both in vitro [33,34] and in the cell [7], can lead to DNA compaction, but the specific roles of crowders such as soluble proteins or polyribosomes remain unclear. Although an educational explanation of the statistical mechanics of DNA is given in [35] (also, see supplementary information in [36]), the depletion theory of Odijk [5] remains difficult to understand. Therefore, we briefly summarize the characteristics of supercoiled DNA self-interactions and DNA–protein cross-interactions from the viewpoint of a biologist.

**Figure 4:**
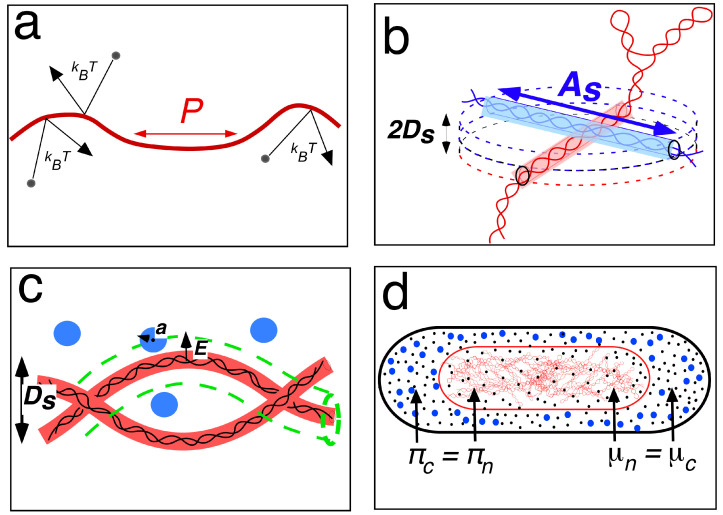
Graphical summary of Odijk’s free-energy approach; also, see Appendix A. (**a**) Topology of the linear double helix with diameter *d_eff_* = 2 nm: solvent molecules (black circles) collide with a linear DNA strand with 1 k_B_T of energy, the amount of energy per thermal fluctuation. The continuous collisions cause the DNA to resemble a wormlike chain. Nevertheless, sections of the chain somewhat less than the persistence length *P* (about 45 nm, giving a Kuhn length of 90 nm) are essentially rodlike. Two points on the DNA contour separated by a distance much greater than *P* are uncorrelated (see Figure 3a and Equation (1) in [35]. (**b**) The DNA double helix is depicted as a branched supercoil. See Appendix A for the calculation of the excluded volume of two colliding superhelical Kuhn segments. (**c**) Electrostatic cross-interactions between soluble proteins (blue circles with radius *a* = 2.3 nm) with the DNA double helix, which has depletion radius *E* = 4.7 nm (dashed green tube). See Appendix A for the calculation of the cross-interaction volume of a protein and the DNA. (**d**) Minimalization of the total free energy is obtained by assuming a phase separation between the cytoplasm and the nucleoid and imposing equal osmotic pressure (π) and chemical potential (μ) in the two coexisting phases (with indices *c* and *n* for cytoplasm and nucleoid).

Based on concepts of equilibrium statistical mechanics [37], polymer physicists regard linear double-stranded DNA as a semi-flexible polyelectrolyte polymer consisting of stiff, freely jointed segments in which chemical details, such as the sequence of base pairs, are not considered. The segments can move relative to each other, and because of the thermal motion that causes the surrounding solvent molecules to bounce continuously against the polymer (with an energy of ~1 k_B_T, the product of the Boltzmann constant and temperature), the elastic DNA rods (with diameter *d_eff_*) undulate and take the shape of a wormlike chain (Figure 4a). However, the chain is resistant to bending, which is reflected by straight segments with persistence length *P*. The chain can thus be viewed as a random walk with a step length longer than *P*, also called the Kuhn length, *A,* a statistical entity with a length of about *2P*. Fluctuations of the long thin chain due to Brownian motion make the Kuhn segments collide with each other, causing the excluded volume effect. Because each chain has a finite volume that is excluded from the rest of the chain, and because a chain cannot pass through itself, the rodlike segments exclude a volume with size *A^2^d_eff_,* representing an expansion or swelling of the long chain (also see Figure 3 in [38]).

These considerations regarding linear DNA also hold for plectonemic supercoiled DNA. Under the bombardment of solute molecules, superhelical DNA will also behave like an elastic structure, causing a strong excluded volume effect through interactions between supercoiled Kuhn segments. We now consider a “superwormlike chain” to have persistence *Ps* and an effective Kuhn length of *A_s_ = 2 P_s_* (Figure 4b).

Equations for the free energy of principal excluded volume interactions between segments of the superhelical DNA itself, and the steric repulsive cross-interactions between the DNA double helix and soluble proteins are discussed in Appendix A. The starting point for the calculations based on Odijk’s depletion theory [5] is obtained by formulating the excluded volumes for both interactions (*B_self_* in Figure 4b and *B_cross_* in Figure 4c). These expressions, scaled by thermal energy (k_B_T) and the total volume of the system (*V_cell_*), give us the free energy of the supercoiled DNA (*F_self_*) and its interaction with soluble proteins (*F_cross_*). For the latter expression, only the high number of small soluble proteins (~10^6^) is considered, while the possible contribution of large polyribosomes is omitted; because they occur in a much smaller number (~8000), their influence on the energy balance, as calculated in Appendix A, was assumed to be negligible [5]. 

The total free energy of the nucleoid *F_nuc_*, when it is dispersed throughout the cell, can be expressed as the sum of three free energies, i.e., *F_cross_*, *F_self_*, and a mixing term, *F_mix_,* to express the electrostatic repulsion between soluble proteins: *F_nuc_* = *F_cross_* + *F_self_* + *F_mix_* (cf. Equation (15) in [5]).

In view of the phase-contrast microscopic observations (Figure 1), it can be assumed that a phase separation exists between the nucleoid and the cytoplasm. By taking the derivative of the above equation with respect to cell volume, we obtain the force for compaction or osmotic pressure, π. By taking the derivative with respect to the number of proteins, we obtain the force for mixing or chemical potential, μ. A minimal free-energy situation or thermodynamic equilibrium is imposed by equalizing these two forces throughout the two phases (Figure 4d). This results in two coexistence equations (see Equations (16) and (17) in [5] and Appendix A). From these two equations and a third equation for the protein volume fraction of total proteins, the protein volume fractions in the cytoplasm (*v_c_*) and nucleoid (*v_n_*) and the volume of the nucleoid (*V_n_*) can be calculated and compared to the experimental values. It should be noted that the full thermodynamic equilibrium assumed here for the calculations does not hold for a living cell, which is in a stationary dynamic (or steady-state) equilibrium.

As shown in Appendix A, interactions between superhelical DNA and soluble proteins (Figure 4c) “overwhelm” the self-energy of the DNA (Figure 4b), leading to the observed phase separation in a minimal energy situation. The results [5,35] indicate that the protein volume fraction in the cytoplasm (*v_c_* = 0.166) is much larger than the protein volume fraction in the nucleoid (*v_n_* = 0.06), as calculated for a nucleoid volume (*V_n_*) of about 0.1. These values explain the observed refractive index difference between the nucleoid and the cytoplasm in phase-contrast microscopy (Figure 1). However, as discussed in the next section, new estimates indicate that the volume of the nucleoid may be much larger (about threefold; see Table 1). 

Here, it should be mentioned that Theo Odijk has re-evaluated the formalism of the equations developed for his 1998 depletion theory, with the tentative conclusion that multiple solutions could be possible. Regarding the effect of polyribosomes (see Section 3.1), they could cause a perturbation not much larger than 10%; he will come back to this problem in future work [39].

### 2.3. Fluorescence Microscopy: New Estimates of Nucleoid Volume

The rare pictures that have been made with a confocal scanning light microscope (CSLM) show an exceptionally small nucleoid with a volume of 0.07 µm^3^ [28]. A slightly larger volume of 0.12 µm^3^ was estimated from an archived original print marked with the original instrumental CSLM magnification. With the development of fluorescence microscopy, several groups have estimated the volume of the nucleoid in slow-growing *E. coli* cells, in which the nucleoid forms a simple rodlike shape. The DNA was either tagged with fluorescent proteins or stained with 4′,6-diamidino-2-phenylindole (DAPI). The results were compared to early measurements of unstained *E. coli* B/r cells [28] obtained with a CSLM, as shown in Table 1.

**Figure 5:**
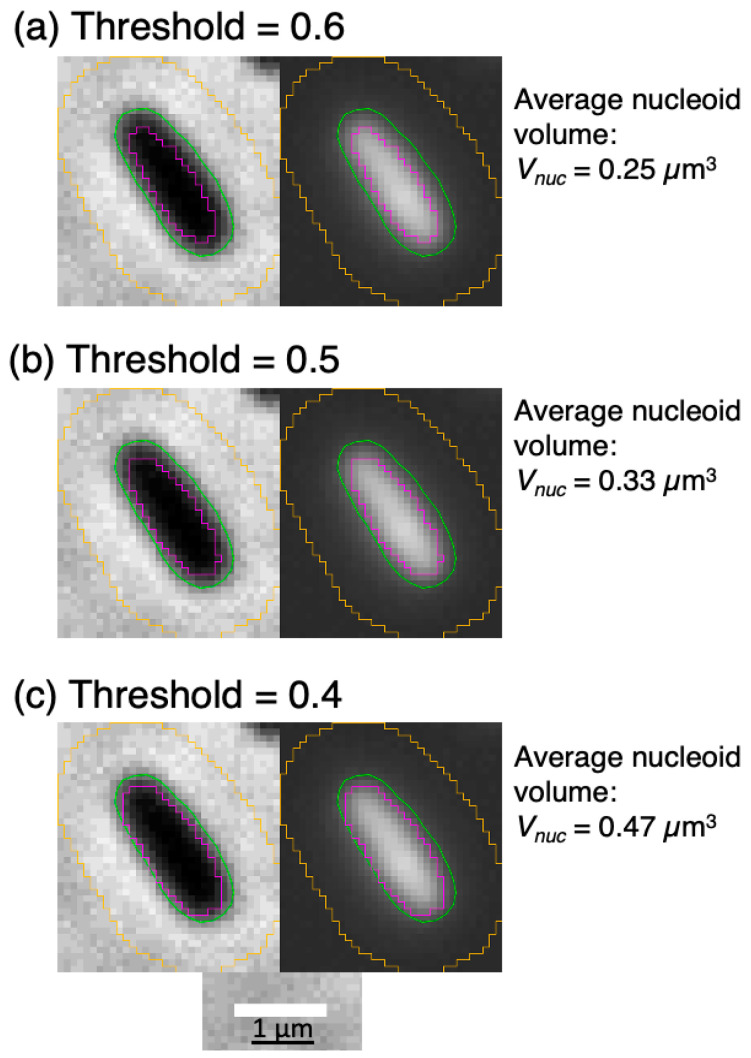
Measurement of nucleoid volume in a population of living *E. coli* B/rH266 cells grown in alanine medium (doubling time 150 min), stained in growth medium with DAPI [40]. Cells and nucleoids were analyzed with ImageJ plugin (Coli-Inspector-04o+NucVol.ojj) at 3 thresholds. The program finds the cell contour in the phase-contrast image (green line in panels) and extends it with 8 pixels (yellow contour). The area of a nucleoid in the fluorescence channel, including its neighborhood, is temporarily smoothed before its min and max values are detected. Then, in the fluorescence images (right panels), an intensity threshold of, for example, threshold = min + 0.4 × (max – min), is applied to define the nucleoid contour using the 3 threshold values indicated (magenta contour). The contour is converted to a “best-fitting rod” in order to measure length and diameter and calculate nucleoid volume. Here, the three values for the average nucleoid volume are given. For the nucleoid volumes of newborn cells, see Table 1, last row. The results of the calculations using the 0.5 threshold best correspond to measurements by eye and hand.

The estimates obtained from fluorescence microscopy images of *E. coli* K-12 cells show a much larger nucleoid volume (0.23–0.27 µm^3^; column 4 in Table 1). Although we look at the nucleoid visualized by light absorption with the CSLM [28,30] and at the light emitted from fluorochrome excitation with the fluorescence microscope, it is unlikely that this difference can explain the different volume measurements. Therefore, we decided to remeasure the images obtained from the same *E. coli* B/r strain grown under similar conditions [40]. As shown in Table 1 (last row), three nucleoid volumes were obtained, depending on the threshold used (Figure 5; see also Table A2 in Appendix B). 

An exceptionally large nucleoid volume (0.7 μm^3^) was measured by Jacobs-Wagner’s group using the Oufti open-source software package [9]. Other estimates by Kleckner’s [41] and Männik’s [7] groups arrived at values of 0.27 and 0.23 µm^3^ for the nucleoid volumes in newborn and early cell cycle cells, comparable to the 0.24 µm^3^ obtained for DAPI-stained cells [40] at a threshold of 0.5 (see Table 1 and Figure 5b). The next section discusses these larger nucleoid volumes and uses them to compare the different polymer physics approaches of Odijk [5] and Männik’s group [6].

**Table 1:** Cell and nucleoid volumes measured from microscopic images of slow-growing live *E. coli* cells. Nucleoid volumes were estimated for cells in the early cell cycle (in *B*-(*G1*-) period) or calculated for newborn cells.

*E. coli* Strain	Doubling Time at 37 °C (Min) ^(1)^	Volume of Newborn Cells (µm^3^) ^(2)^ (Cell Number Measured)	Nucleoid Volume in Newborn Cells (µm^3^) (Threshold)	DNA Concentration in Nucleoid ^(3)^ (mg/mL)	Microscopy/Staining: Figure(s) in References
B/rH266	150	0.33 (10)	0.07 0.12 ^(4)^	69 40	CSLM/unstained: Figure 1 in [28]
K-12 (NK9387)	~70 (125 at 30 °C)	0.33 (2)	0.27 (0.5)	18	Fluor. microscopy/HupA-mCherry: Figures 1B and 3B in [41,42]
K-12 (MG1655)	110 (220 at 28 °C)	0.45 0.50 ^(5)^	0.23 0.25	21 19	Fluor. microscopy/HupA-mNeonGreen: Tables S2 and S5 in [7] Fluor. microscopy: Figure 2 in [6]
K-12 (CJW6324)	81 ^(6)^	- (B-period cells) (*n* = 19,510)	0.7	7	Fluor. microscopy/DAPI: Figure 7A in [9]
B/rH266	150	0.58 (281)	0.18 (0.6) ^(7)^ 0.24 (0.5) 0.34 (0.4)	27 20 14	Fluor. microscopy/DAPI: Figure 5 in main text [40]

^(1)^ Doubling times refer to growth at 37 °C. Growth at 28–30 °C is assumed to be 2× slower. ^(2)^ The volume of newborn cells is obtained by dividing the volume of the average cell (Vav = 0.81 µm^3^) by 2ln2, assuming exponential growth of an individual cell. ^(3)^ The amount of DNA in non-replicating chromosomes is 4.8 × 10^−12^ mg [43]. The amount of DNA in replicating cells with average length and volume is calculated from average chromosome equivalents (genome content) per cell using the expression given by Cooper and Helmstetter [44]: Gc = Td/Cln2 (2^(C+D)/Td^ – 2^D/Td^). Under slow growth, a newborn cell in the B-(G1-) period is assumed to contain 1 chromosome equivalent. ^(4)^ Re-estimated using instrumental magnification of 23850× as indicated on the original photographic print. ^(5)^ Calculated from average cell *Vcell* = 0.7 µm^3^; *Vnewborn* = 0.7/2ln2 = 0.5 µm^3^. See note (2) and Table A1. ^(6)^ Doubling time from Figure 1B in Gray et al. [45]. ^(7)^ The DAPI-stained population of [40] was remeasured at different thresholds using ObjectJ plugin of Vischer: Coli-Inspector-04k+NucVol(0.5).ojj. See caption for Figure 5 and Table A1 in Appendix A. The average cell length of the population (cell count 607) is 2.31 µm, with a cell diameter of 0.46 µm. The average length of the nucleoid is 2.31 µm, with a diameter of 0.46 µm.

## 3. Different Views on DNA Compaction

### 3.1. Compaction through Polyribosome Exclusion

In his analysis, Odijk [5] only considered the high number of soluble proteins (~10^6^ per cell) because it was assumed that larger crowder particles, present in a relatively small number like ribosomes (8000 in slowly growing cells), would exert a negligible influence on the energy balance. However, later studies proposed that crowders like polyribosomes might have an effect on nucleoid compaction because of their larger size. Ha’s [3] and Männik’s [7] groups adopted Odijk’s free-energy approach while taking into account not only the numbers of crowders but also their size. It should be noted that the nucleoid compaction discussed here is the effect of steric interactions between crowders (proteins and polyribosomes) and DNA. A different view, discussed in Section 3.2 below, is that nucleoid compaction is not caused by steric interactions between DNA and crowders but by the poor solvent quality of DNA in the cytoplasm.

In the study by Männik’s group [7], they measured the compaction of nucleoid volume by decreasing the volume of the cell by increasing the osmolality of the suspension medium (hyperosmotic shock with NaCl) or by mechanically squeezing cells in a microfluidic chip in which pressure could be applied. Their observations showed that a 30% increase in crowder concentration can cause a threefold decrease in nucleoid volume in living cells. To measure nucleoid volume, they used small cells in the early stage of the cell cycle because these contain nucleoids with a simple ellipsoidal shape that can be measured relatively easily (see Table 1, row 4, columns 3 and 4).

**Figure 6:**
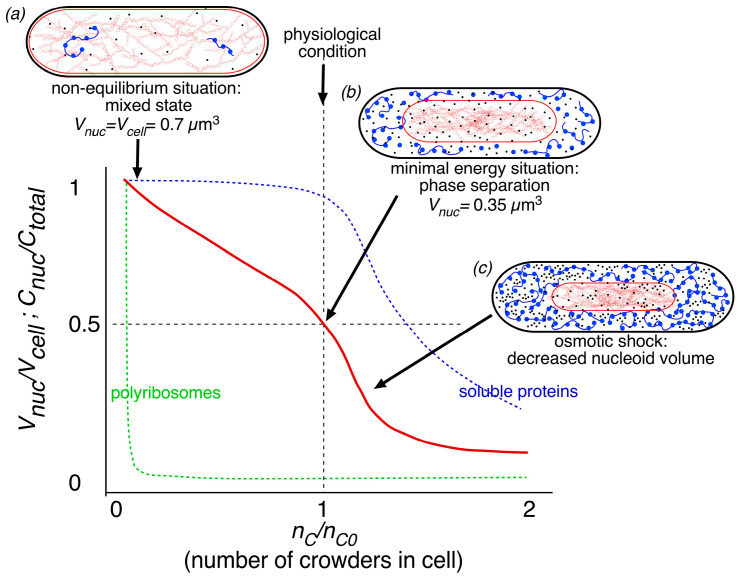
Schematic qualitative representation of a nucleoid compaction curve comparable to Figure 2a in [6]. The solid red line shows the nucleoid volume relative to cell volume as a function of the number of crowders (polyribosomes or proteins). *n_c_* indicates either polyribosomes or proteins, and *n_C0_* is the corresponding number under normal physiological conditions. The dashed green and blue lines represent the relative crowder concentrations within the nucleoid for polyribosomes and soluble proteins, respectively. Cell *(a)* is a model cell (fixed volume of 0.7 µm^3^) in a non-equilibrium situation with a fully dispersed nucleoid and a homogeneous crowder mixture of small proteins and large polyribosomes. From left to right, an increased relative polyribosome concentration shows immediate phase separation (see inset of Figure 2a in [6]) and a full exclusion of polyribosomes from the nucleoid (dashed green line). A slow compaction of nucleoid volume (solid red line) is obtained upon increasing the polyribosome concentration. Cell *(b)* shows the equilibrium situation at physiological concentrations of polyribosomes and soluble proteins. Nucleoid volume decreased to half the cell volume (*V_nuc_* = 0.35 µm^3^), while soluble proteins still occurred at ~95% within the nucleoid volume (dashed blue line). Cell *(b)* was also used for comparison in Figure 7a. Cell *(c)* shows that upon a further increase in crowder concentration (e.g., by osmotic shock), the soluble proteins become depleted from the nucleoid and nucleoid volume rapidly decreases.

In an elaborate theoretical follow-up of the experimental work of Yang et al. [7], Männik’s group further investigated the contributions of crowder species, including soluble proteins as small crowders (diameter of 5 nm) and polyribosomes as large crowders (radius of gyration: 35 nm), interacting with supercoiled DNA (see Figure 1 in [6]). Their work is summarized here in the nucleoid compression curve of Figure 6. The curve shows that rapid exclusion of polyribosomes from the nucleoid (dashed green line) generates a phase separation between the cytoplasm and the nucleoid (solid red line) in the transition from cell *(a)* to cell (*b*) in Figure 6. The solid red line shows that under normal physiological conditions, about 95% of the soluble proteins are still present in the nucleoid (dashed blue line). Only upon a further increase in the concentration of small crowders was a decrease in nucleoid volume obtained, accompanied by a depletion of soluble proteins from the nucleoid in the transition from cell *(b)* to cell *(c)* in Figure 6. 

The proposal of Männik and coworkers that polyribosomes are the dominant factor in establishing a phase separation between the cytoplasm and the nucleoid is based on considering hypothetical cells in which only polyribosomes are present. The compaction curve of these simulated cells initially shows the same phase separation as when polyribosomes are present together with proteins. However, with only polyribosomes as crowders, the relative nucleoid volume (*V_nuc_/V_cell_*) decreases no more than ~0.4 (Figure 2b in [6]). In addition, the simulation shows that when only proteins are present, a mixed state of the nucleoid and proteins is maintained (as in cell (*a)* in Figure 6). Phase separation (cell *(b)* in Figure 6) now only occurs at a relative high protein number when *n_c_/n_c0_* > 1.4 (cf. Figure 2c in [6]). In contrast to the dominant effect of large polyribosomes (radius of gyration: 35 nm), Cunha et al. [34] observed that small polyethylene glycol molecules (radius of gyration: ~1.5 nm) were effective in compacting nucleoids isolated from *E. coli*.

Männik group’s theoretical approach makes different polymer physics assumptions and uses different input values than the work of Odijk [5]. This complicates comparing the two studies. Odijk [5] obtained energy minimalization by equalizing the chemical potentials and osmotic pressures in the nucleoid and cytoplasm (Figure 4d), as explained in Appendix A. Chang et al. [6] obtained minimization of total free energy of the cell by using best-fit values for parameters *g* and *a* in Equation (2). By applying this equation, as formulated by Cunha et al. [34], the free energy of DNA self-interactions *F_self_* includes the cross-linking of DNA in the nucleoid. As a result, the value of *F_self_* is somewhat lower (0.58 × 10^4^ *k_B_T*) than the value (1.9 × 10^4^ *k_B_T*) obtained by Odijk [5], who considered only the excluded volume interaction between the supercoiled Kuhn segments.

Furthermore, Chang et al. [6] used different input values for supercoil persistence length (50 nm versus 80 nm in Odijk’s model), supercoil contour length (493 µm versus 630 µm), and number of supercoiled (Kuhn) segments (*N_s_* = 6700 versus 4000 estimated by Odijk [5]). In addition, they used different input values for ribosomes (6000 versus 8000) and total number of soluble (non-ribosomal) proteins in the cytoplasm and nucleoid (0.23 × 10^6^ versus 1.6 × 10^6^) (see Table A2 in Appendix B); this sevenfold difference could have had a great impact on their respective conclusions. In the work of the Männik group [6], the number of soluble proteins was again obtained by fitting the model to the experimental data (see Figure 1c in [6]), whereas Odijk’s group obtained the value from the physiological measurements of Bremer and Dennis [46].

**Figure 7:**
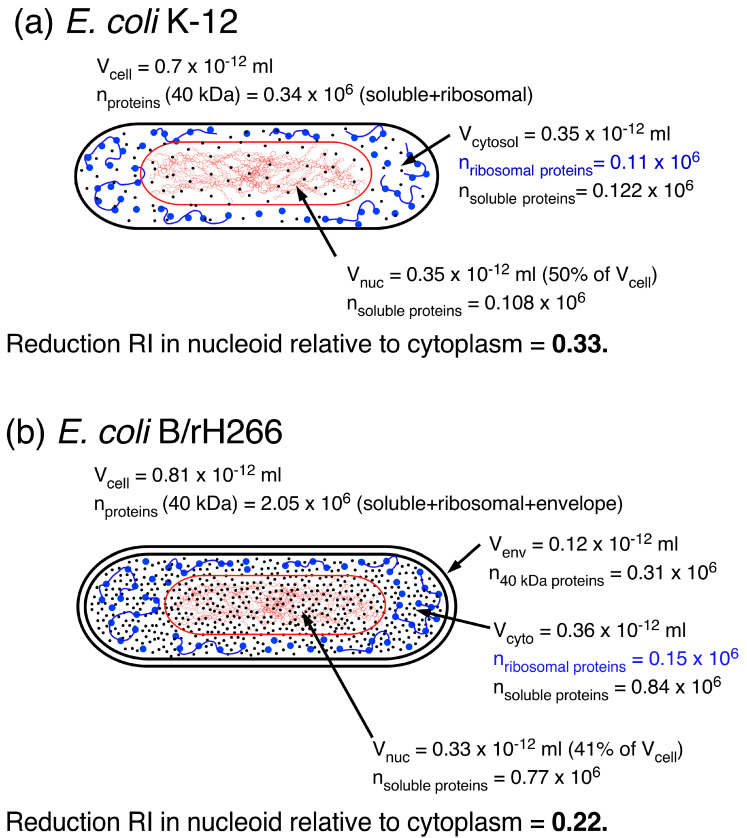
Schematic representations (not to scale) of (**a**) an average *E. coli* K-12 cell from [6] and (**b**) an average *E. coli* B/rH266 cell [40] (see Table 1 and Figure 5b). The cell in (**a**) is the same as cell *(b*) in Figure 6. The cell in (**b**) is from the same population as the cell in Figure 5. For both cells, it is assumed that polyribosomes are excluded from the nucleoid, whereas soluble proteins can diffuse throughout the cytoplasm and nucleoid and are thus not depleted from the nucleoid, as proposed by Odijk [5]. For the calculated reduction of the RI of the nucleoid relative to the cytoplasm, see Appendix B, Table A2, note (12). For the measured dimensions of the cells in (**a**), see [6], and for the cells in (**b**), see Table 1, note (7).

Männik and coworkers [6] also emphasized that “the experimental uncertainties for several parameters entering the expression of the total free energy are considerable”. This certainly holds for the volume of the nucleoid (see Table 1) and the number of soluble proteins assumed to be present in slow-growing *E. coli* cells (see Figure 7). These are the parameters that determine the density and thus, the RI values of the nucleoid and cytoplasm, as discussed in Section 2.1. If phase separation is only the effect of the exclusion of polyribosomes, and if soluble proteins will diffuse throughout the cell, what protein concentration difference and thus, RI difference between the cytoplasm and the nucleoid can be expected? To evaluate this question, we compared cytoplasmic and nucleoid volumes and numbers of ribosomal and soluble proteins between average *E. coli* K-12 cells (Figure 7), as described in [6], and remeasured *E. coli* B/r cells [40], both presented in Table 1. A detailed description of the relevant assumptions and data is given in Appendix B, Table A2. The comparison (Figure 7a) shows that for the cells in [6], a 33% reduction in the RI value of the nucleoid relative to the cytoplasm was obtained. This is in agreement with the reduction observed in [28], as described in Section 2.1. For the cells in [40], the RI reduction was only 22% (Figure 7b), which can be ascribed to the assumed higher protein concentration in these cells. This suggests that given the assumptions made for this cell, an additional depletion of soluble proteins from the nucleoid to a concentration of ~13 mg/mL and an increase in protein concentration (soluble and ribosomal) in the cytoplasm to ~20 mg/mL would be needed to obtain an RI reduction in the nucleoid of about 34% (see Table A2 in Appendix B). 

The total volume fraction of soluble proteins in the cells in [6] calculated from Figure 7a is 0.017; the protein volume fraction calculated for the cells in Figure 7b is 0.101. Future estimates and measurements will have to be made to show whether the sevenfold lower input value for soluble proteins used in [6] is realistic.

### 3.2. Compaction through Poor Cytoplasmic Solvent Quality or Transcriptional Activity

Section 2.2 and Section 3.1 described DNA compaction based on free energy minimization of steric DNA self-interactions and DNA–protein cross-interactions. A different approach was taken by Jacobs-Wagner’s group [9] based on the solvent quality of chromosomal DNA in the cytoplasm. 

As a starting point, Xiang et al. [9] considered DNA to be a “random-coil polymer” (see Figure 1A in [9]), in which compacted DNA chains contact each other and form a network with cross-points depending on the DNA concentration and the quality of the cytoplasmic solvent, consisting of water, proteins, ribosomal subunits, and polyribosomes. The distance between cross-points, i.e., correlation length *ξ*, determines the mesh size of the network. They calculated the solvent quality using an equation derived by Rubinstein (see reference in [9]). Important parameters for this equation are a Kuhn length for the double helix of 60 nm (rather than 90 nm, as given in Figure 4a), a DNA concentration in the nucleoid of ~7 mg/mL, which is about 3× lower than the value obtained in other studies (see Table 1), and an average mesh size of 50 nm. This last value was determined experimentally using probes (GFP-µNS particles) varying from 50 to 150 nm. The observations showed that the apparent average mesh size in the nucleoid must be around 50 nm (see Figure 2C,F in [9]). With the above input values, the Flory exponent was calculated. This exponent indicates solvent quality and had a value of *ν* = 0.36. Such a low value (<0.5) suggests that the cytoplasm acts as a poor solvent for DNA. Here, it should be emphasized that in contrast to the branched DNA superhelix models used by Odijk ([5]; see also Figure 5 in Wegner et al. [47]) and Männik and coworkers ([6]; see also Figure 3B in [35,48]), Jacobs-Wagner’s group only considered circular, non-supercoiled DNA for their calculations and poor solvent simulations (see Figure 3 in [9]). 

The results of their experiments on the spatial distribution of (poly)ribosomes and the effect of transcription inhibition by rifampicin, causing expansion of the nucleoid (see below), prompted the authors to suggest that RNAs in general cause an effective poor solvent quality for DNA in the cytoplasm. These results [9] agree with recent super-resolution and single-molecule fluorescence microscope studies (see review in [49]), suggesting that large proteins [50] and free ribosomal subunits (~20 nm diameter) are able to diffuse through the nucleoid [51,52,53]. These microscopic observations suggest that soluble proteins and larger particles (up to 20 nm diameter) are not depleted from the nucleoid and that the nucleoid thus contains a heterogeneous mixture of DNA and cytoplasmic components (as suggested by the poor solvent simulations shown in Figure 3 in [9]). 

A recent study by Bignaud et al. [11] describes a different mechanism of DNA compaction (for reviews, see [19,54]). Based on high-resolution chromosome conformation capture (Hi-C) analysis, Bignaud and coworkers propose that the folding of the chromosome is obtained by transcription-induced supercoiled regions in association with SMC proteins. They suggest that transcription-insulating domains (TIDs), which tend to contact each other and cluster together, fold the chromosome and function as its primary building blocks (see Figure 4l in [11]). Their contact maps reveal a succession of short, transcription-induced compact domains alternating with unstructured, highly expressed gene regions that inhibit supercoil diffusion. Previously, Lioy et al. [55] identified ~30 chromosome self-interacting domains (CIDs) with an average size of 150 kb. As expected, the Hi-C patterns induced by transcriptional activity disappeared upon inhibition by rifampicin (Figure 1b in [11]).

It is evident that the inhibition of transcription initiation by rifampicin, which causes the dissociation of polyribosomes and 70S ribosomes and the degradation of mRNA, must have a profound effect on the transcriptional units, as described by Bignaud et al. in Figure 4l [11]. If these transcriptional units play a role in chromosome folding, rifampicin would be expected to annihilate the compaction of the nucleoid. Indeed, rifampicin was found to change the appearance of the nucleoid in the microscopic studies listed in Table 2. Because the growth rate [6] and fixation procedure [54] have been suggested to influence nucleoid appearance, they are also mentioned in Table 2. In 7 of the 18 studies, full dispersal of the nucleoid is noted, all in fast-growing cells; in 2 of these studies, the cells were fixed (see columns 2 and 3 in Table 2), while in the other studies, the changed appearance was described as a radial contraction combined with a longitudinal expansion in both fast- and slow-growing cells. An explanation for this behavior was proposed by Mondal et al. [56] based on computer simulation studies: polyribosomes are preferentially localized at the endcaps of the cell, where the nucleoid becomes compressed axially. When rifampicin causes their dissociation, the nucleoid will expand along the long axis, while the ribosomal 30S and 50S subunits form a thicker layer along the cylindrical wall, compressing the nucleoid radially. Some studies [51,57,58] seem to confirm this explanation, which suggests that there is still a depletion of ribosomes and proteins from the nucleoid, as described by the depletion theory of Odijk in Section 2.2 [5].

When authors suggest that the nucleoid becomes fully dispersed (indicated in the last column of Table 2 with “yes”), it seems plausible that dissociated ribosomal subunits and soluble proteins fully penetrate the nucleoid, annihilating any phase separation between the cytoplasm and the nucleoid ([6]; see Figure 7a). This would imply that phase-contrast microscopy of rifampicin-treated cells immersed in a high-refractive-index medium (see Figure 1a–c) would show no region in the cell with a lower refractive index. Future microscopic studies will have to be carried out to solve the disagreements, evident from the studies listed in Table 2.

The heterogeneous nucleoid structure proposed by Xiang et al. [9] based on poor solvent simulations and by Bignaud et al. [11] should comply with the density difference visualized by phase-contrast microscopy (Figure 1) and must be understood in terms of the polymer physics and thermodynamic rules of equal osmotic pressure and chemical potential in the two phases, as mentioned in Figure 4d. The same holds for the proposal of Bakshi et al. [64] that dissociated 30S and 50S ribosomal subunits can mix with DNA, causing nucleoid expansion. 

## 4. Segregation and Movement of Chromosome Arms (Replichores)

When studying the necessary movement of daughter chromosomes and their left and right replichores, the physical properties of nucleoid compaction described in the previous sections have to be taken into account. This includes properties such as the permeability of the nucleoid to macromolecules involved in transcription and translation. Does the segregation process take place in a heterogeneous region where small and large protein complexes (polyribosomes) are mixed with the nucleoid, as suggested in some fluorescence microscope studies [51,52,53]? Or does segregation occur in a relatively homogeneous nucleoid, where small soluble proteins are depleted to some extent from the nucleoid, as proposed by Odijk ([5]; see also [35])? As described by Kohiyama et al. [67], the replication bubble starts with a DnaA-based hyperstructure that integrates the many proteins involved in the necessary metabolic and regulatory pathways for the initiation of replication. Here, however, only the structural aspects of the nascent chromosome arms and their behavior during segregation are considered. 

### 4.1. Replichore Movement to Opposite Halves of the Nucleoid

**Figure 8:**
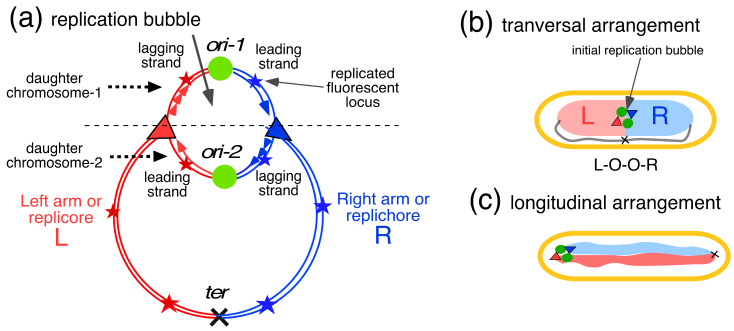
Schematic representation of the circular chromosome and localization of replichores in the nucleoid. (**a**) Left and right chromosome arms (replichores) with replicated origins (green circles) in the replication bubble. During the replication or *C*-period [72], the arms are replicated by the two replisomes (red and blue triangles), each synthesizing a leading strand and a lagging strand with Okazaki fragments (short triangles). Several fluorescently labeled loci (red and blue stars) are indicated, showing the position of the loci in the growing cells from Flemming Hansen (see below). The two daughter chromosomes (dashed arrows) end up in the prospective daughter cells with their replichores in two opposite halves of the nucleoid. The origin localizes at mid-cell between the left and right replichores that are connected by the terminus (black cross). (**b**) Position in a slow-growing *E. coli* cell (cf. Figure 5), with the two chromosome arms in the transversal arrangement. Initiation of DNA replication at mid-cell forms a replication bubble, resulting in a replichore pattern indicated as Left-ori-ori-Right (L-O-O-R). The terminus occurs in a stretched connection between replichores and migrates after duplication and cell division from the new pole to the cell center. (**c**) For comparison, a longitudinal arrangement of chromosome arms (cf. *Caulobacter* [17] or *Agrobacterium* [18]). The replication bubble is positioned at the cell pole; the terminus is at the other tip of the nucleoid [17].

As depicted in Figure 8a, the circular chromosome of *E. coli* can be divided into two chromosome arms, called left (*L*) and right (*R*) replichores, that run from the origin to the terminus. Bi-directional replication from the origin results in a replication bubble at initiation, in which two nascent pairs of replichores (red and blue), connected by an origin, represent the two daughter chromosomes. These then segregate into the two prospective daughter cells. Studying the paths of fluorescent loci in slow-growing *E. coli* cells through time-lapse experiments, the groups of Sherratt [13] and Hansen [14,68] found that the *E. coli* chromosome is arranged with its left and right replichores lying separately in opposite halves of the nucleoid (Figure 8b). Their important observations and views (for reviews, see [69,70,71]) form the basis for the discussion below and for the speculative proposal of a passive segregation model in Section 4.2. 

Using the constructs of Flemming Hansen, in which the origin and two loci on the left and right replichores were tagged with three different colors, the observations of Wang et al. [13] and Nielsen et al. [14] could be confirmed by measuring the simultaneous movements of the three loci [66].

**Figure 9:**
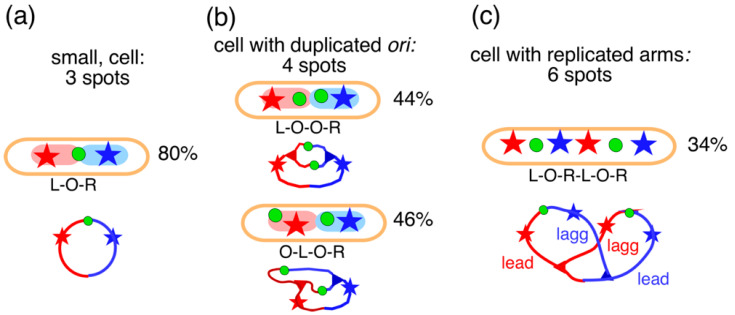
Measurement of positions of origins (green circles) and fluorescently labeled foci (red and blue stars), as observed in the constructs of Flemming Hansen in slow-growing *E. coli* cells with a doubling time of 150 min at 32°. The diagrams below the cells show the presumed replication status of the chromosome. The percentages indicate the major patterns observed; see tables in [66]. (**a**) Newborn cells with unreplicated DNA. (**b**) Cells with duplicated origins (4 spots) showing 2 patterns. (**c**) Cells with an almost replicated chromosome (6 spots) showing the most common pattern (34%) that, upon division, leads to the pattern (L-O-R) in newborn cells, as shown in (**a**).

The measurements showed that in newborn cells that had not yet initiated replication, the three loci occurred in the typical pattern of L-O-R in 80% of the cells (Figure 9a). This high percentage can be interpreted to indicate that the two replichores had not mixed but ended up separated in the two halves of the nucleoid (see Figure 8b and Figure 9a). If the replichores had intermingled, the three loci would have occurred in three patterns (L-O-R, L-R-O, and R-L-O) and the percentage of the L-O-R pattern would only be 33%. After initiation and origin duplication, the cells contained four spots showing the patterns L-O-O-R (44%) and O-L-O-R (46%). In the majority of four-spot cells with increased length (L >2.3 µm), the origins moved apart and passed either one replichore locus (36%; O-L-O-R/O-R-O-L; Figure 9b) or both replichore loci (26%; O-L-R-O). This indicates that soon after duplication, one of the origins passed an unreplicated locus on one of the replichores (see also Table 1A in [66]). Figure 9c shows a pattern in cells with six spots indicative of an almost fully replicated chromosome. As indicated by the schematic chromosome image in Figure 9c, the asymmetric L-O-R-L-O-R arrangement is obtained when the leading strands move faster (perhaps through transcriptional activity) rather than the lagging strands. This behavior was described by Mäkelä et al. (see Figure 5A in [73]), who attributed it to the primarily MukBEF-dependent binding of DnaN (*β2*-clamps) to the lagging strands.

**Figure 10:**
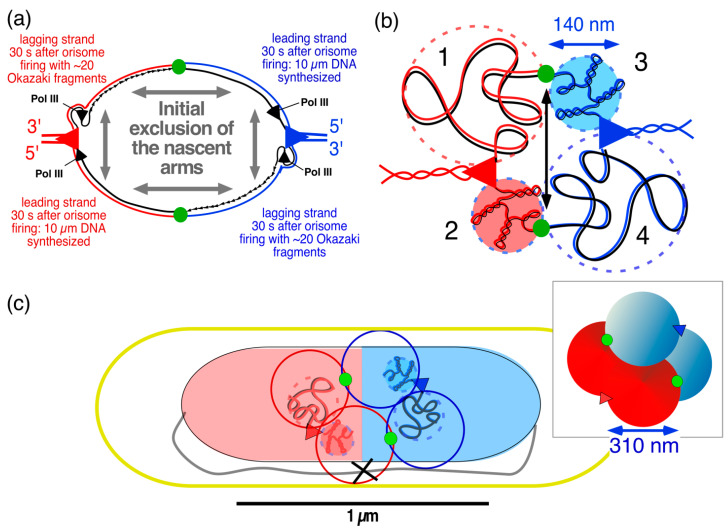
The four-excluding-arms model: daughter strand exclusion in the initial replication bubble (orisome). (**a**) Schematic drawing of the initial stage, 30 s after the firing of the origin (green circles). Leading and lagging strands of ~10 µm synthesized by the two Pol III replicases of each replisome (red and blue triangles. See Appendix C). The nascent leading and lagging arms are proposed to exclude each other (remain de-mixed) because of physical differences (random coil versus supercoiled segments) and physiological differences (transcription of different genes). (**b**) Initial stage of the replication bubble 30 s after initiation: the 2 leading chromosome arms (#2 and #3) will immediately become supercoiled and fold into a blob (diameter of 140 nm; see Table A3). The 2 lagging arms (#1 and #4) will adopt a random coil conformation until the Okazaki fragments (about 20 fragments) are ligated. Due to the tethering of the two replisomes to the parental DNA they are replicating, the enlarging blobs push the origins apart (double arrow) (see also description of Figure 4 in [8]). (**c**) A 2D projection of the developing microdomains: after 5 min, nascent chromosome arms of 100 µm of DNA are synthesized and enlarged into microdomains with a diameter of 310 µm. The inset shows a 3D representation. Red and blue circles represent nascent microdomains of left and right replichores; black cross represents the terminus (compare with Figure 8a). Further enlargement of the microdomains by DNA synthesis will force these domains to rearrange their positions in the narrow tube of the nucleoid (see Figure 11(b1) and Table A3 in Appendix C).

**Figure 11:**
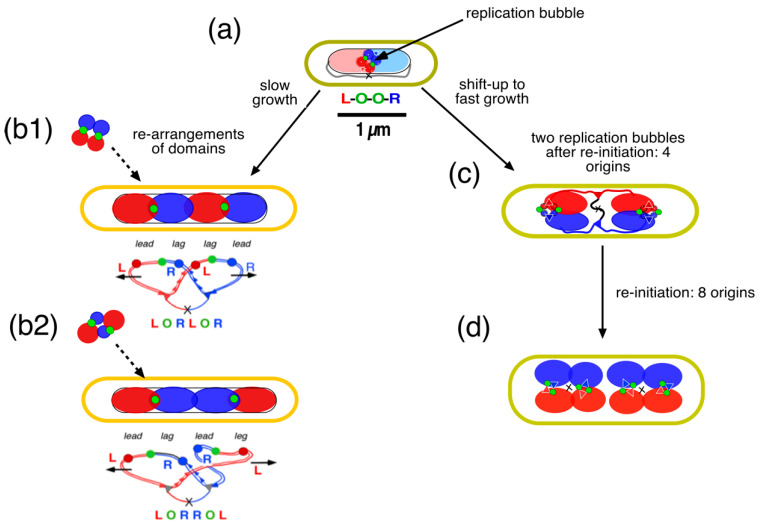
Schematic representation of the development of 4 excluding microdomains in the transversal arrangement of *E. coli* B/r during slow growth (*Td* = 150 min) and longitudinal arrangement after shift-up to fast growth (modification of Figure 2 in [91]). (**a**) The 4 initial microdomains, as depicted in Figure 10b,c, are drawn as a tetrahedron of 4 spheres (not to scale) connected to each other by the origin (green circle) and the feeding threads emanating from the replisomes replicating the parental DNA (light colored regions). In this 4-spot cell (cf. Figure 8b), the pattern is L-O-O-R. (**b1**) At the end of replication, the 4 spherical domains, enlarged through continued DNA synthesis, rearranged themselves in the long axis of the narrow nucleoid as indicated by the dashed arrows. They end up in a transversal arrangement. Because both leading arms of the two replichores move faster than the lagging arms, the asymmetric ordering pattern L-O-R L-O-R is obtained. (**b2**) An alternative arrangement can be obtained because of a faster expansion of both the leading and the lagging arms of the red replichore (caused by pausing of the blue replisome), resulting in the symmetric pattern L-O-R R-O-L (see [69]). (**c**) Nutritional shift-up from a doubling time of 150 min to fast growth (*Td* = 24 min). After 60 min, the average cell is wider and contains 4 origins after reinitiation. Because the microdomains do not have to rearrange in the wider cell, the origins remain at the tip of the nucleoid in a longitudinal arrangement. (**d**) After 90 min, the average cell contains 2 nucleoids with a total of 8 origins (for the construction of the DNA replication cycle during shift-up, see the cell cycle simulation program in [92]). Cell sizes reflect the measurements of *E. coli* B/r cells by Woldringh et al. [93]. See also Youngren et al. [76] for the longitudinal arrangement of the *E. coli* nucleoid in fast-growing cells.

To obtain the various patterns in the transversal arrangement, the replicating left and right arms have to pass each other once or twice. As will be explained below (Section 4.2, Figure 10 and Figure 11), the formation of these patterns and thus, the mechanism of movement of loci or arms, results from the development, in the initial replication bubble, of replichore domains that do not become mixed. These separate domains within the nucleoid are assumed to enlarge and displace each other through continued DNA replication. 

It should be emphasized here that, except for the origin (Figure 9b), the occurrence of three adjacent spots (L-L-O-O-R-R) was never observed. This can be interpreted to indicate that replichore loci separate almost immediately after replication (see also the discussion on cohesion in Section 4.3). This contrasts with unpublished measurements of *E. coli* FH4035 cells (see construct of Flemming Hansen in [66]) grown in LB medium at 30 °C (doubling time of 66 min) and treated with 30 µg/mL nalidixic acid for 2–3 generations: the short filaments showed duplicated colocalized spots, mostly separated in the short axis, suggesting that de novo DNA synthesis is required for segregation in the long axis of the cell.

Because it has been suggested that the active process of transertion [74] may play a role in the movement of segregating daughter strands, experiments were performed with cells treated with 300 µg/mL rifampicin, which inhibits transcription and growth but allows run-off DNA synthesis and residual division [66]. If replicated spots did not segregate in rifampicin-treated cells, we would expect to observe a high percentage of cells with adjacent spots (L-L-O-O-R-R). However, as can be seen from Table 3, the replichore patterns found in non-growing cells were similar to those in control cells. In view of (i) the significantly decreased percentage of cells with four or five spots resulting from continued run-off replication (Table 3, column 4), (ii) the absence of cells with adjacent LL-OO-RR spots, and (iii) the decreased percentage of cells showing the origin lying outside of the other loci (Figure 9b; see Table 2 in [66]) in both growing and non-growing cells, it was concluded [66] that chromosome movement in non-growing cells occurs in a similar way as in growing cells: in both cases, the two arms migrate to different halves of the nucleoid with the origin in between. This result contradicts a previous proposal that the process of transertion drives DNA segregation [74] and supports the hypothesis that segregation is passively driven by the process of de novo DNA synthesis. The idea of replication-driven segregation has been proposed by the groups of Grossman [75], Hansen [14,68], Sherratt [13], Austin [68,76], and Wiggins [77]. A direct link between DNA replication and chromosome organization has been demonstrated by the Sherratt group [69] and will be discussed in the next section. 

**Table 3:** Summary of ordering patterns in growing and non-growing cells as documented in Tables 1A, B, 2, and 3 in [66]. A fully random ordering pattern would result in percentages of 50, 25, and 25 in columns 7 to 9, respectively.

Strain FH4035 ^(1)^	Number of Sequences Analyzed	Average Number of Spots/Cell	% Cells with 4–5 Spots	% Cells with 6 Spots	Average Length of 6-Spot Cells (µm)	% R-L-R-L/ L-R-L-R ^(2)^ 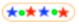	% R-L-L-R ^(2)^ 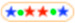	% L-R-R-L ^(2)^ 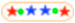	% L-L-R-R
Control (average of 8 experiments)	4072	4.2	13/5	39	3.02	64	19	17	3
Rifampicin treatment ^(3)^ (average of 3 experiments)	2848	4.6	1/1	53	2.73	51	28	21	0

^(1)^ In this strain, the loci on the two replichores become replicated 30 min after the initiation of replication. ^(2)^ Green circles represent the origins; the blue and red stars represent fluorescently labeled foci. See the captions for Figure 8 and Figure 9. ^(3)^ Cells were treated with 300 µg/mL rifampicin for 210 min at 28 °C.

Wang and Sherratt [78] presented evidence that inhibiting transcription with rifampicin did not affect the segregation of the origin in *E. coli*, in agreement with the results presented in Table 3. The conclusion of these experiments [66] is that segregation continues during run-off DNA replication, but with a more random ordering of replichores (see columns 7–9 in Table 3). With respect to the distances measured between loci pairs (LL, OO, RR) in (unfixed) rifampicin-inhibited cells, it can be noted that these were about 0.3 µm smaller than in the growing control cells (Table 3 in [66]). This result can be ascribed to the smaller average length of rifampicin-treated cells (column 6 in Table 3), partly due to inhibited elongation and residual division (see Appendix C, Figure A2). 

**Figure A1:**
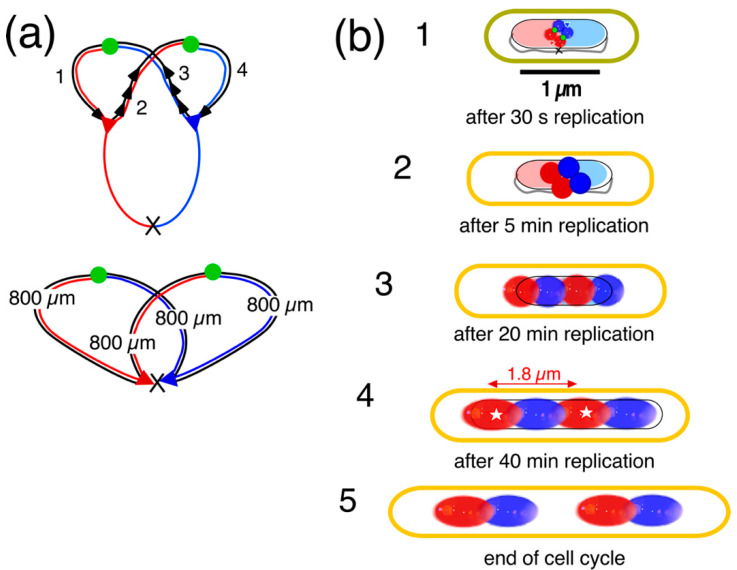
Enlargement and rearrangement of nascent domains in the four-excluding-arms model. (**a**) Schematic representation of the 4 nascent excluding arms (cf. Figure 8a in main text). (**b**) For the volumes of the domains, see Table A3. Sizes (yellow contour) of the *E. coli* B/r cells are not to scale. The replication period is assumed to be C = 40 min. (**b1**) After initiation of DNA replication, the 4 excluding arms in the replication bubble are assumed to form a tetrahedron within the parental nucleoid. (**b2**) After 5 min of replication, the enlarged tetrahedron exceeds the diameter of the parental nucleoid, forcing the domains to rearrange in the long axis. (**b3**) After 20 min of replication, the rearranged domains have formed the most common ordering pattern, L-R-L-R, in the elongating nucleoid (see Table 3 in the main text). (**b4**) After 40 min of replication, the chromosome is assumed to have fully replicated. The two replichore domains of each daughter arm now occupy opposite halves in the developing daughter nucleoids, as observed in [13,14]. The double red arrow indicates the distance over which a replicated locus on the left replichore has segregated. Compare to segregation distances shown for a different strain (*E. coli* K-12) in Figure A2a. (**b5**) Schematic representation of a cell at division, in which the daughter nucleoids have moved apart through continued cell elongation before cell division.

Regarding the active process of transertion, it should be noted that the separation of daughter nucleoids after the termination of replication is dependent on cell growth (Figure A1; see also Figure 3 in [66]). Active processes such as transertion and cell constriction may thus be involved in stimulating this second step of the segregation process, the separation of daughter nucleoids.

### 4.2. Four-Excluding-Arms Model for Segregation [79]

It is tempting to assume that the movement of replicated chromosome arms (Figure 8a) to opposite halves of the daughter nucleoids (Figure 8b) is only possible if the four newly synthesized DNA arms do not become mixed or entangled but are maintained as separate entities from the start of the replication–segregation process. This de-mixed state can be achieved with the help of an active enzymatic mechanism such as energy-consuming (motor) proteins and topoisomerases. These proteins include ATPases such as topoisomerase IV and SMC complexes such as SMC-ScpAB in *Bacillus subtilis* and *C. crescentus*, the MukBEF complex in *E. coli*, and the MksBEF complex in a wide range of other bacterial species [80]. It is generally believed that *E. coli* DNA is organized around an axial core formed by MukBEF complexes, which promote the individualization of chromosome arms and linear compaction of the chromosome through loop extrusion [20,81]. Sherratt and coworkers also proposed that a linear MukBEF axial core could direct the asymmetric (L-R-L-R) segregation of replichores (see Figure 9c) at the replication fork through differential binding of *β2*-clamps to lagging strands (see Figure 5A in [73]). Previously, Jun and Mulder [82] proposed that asymmetric constraints of leading and lagging strands may cause the formation of different patterns during their segregation.

A simpler mechanistic explanation for the different segregation patterns of replichores is given by the four-excluding-arms model, as proposed here and illustrated in Figure 10 [79]. It is hypothesized that chromosome arms containing nascent strands synthesized by the four Pol III replicases (indicated in Figure 10a) become separated by physical exclusion because of topological and physiological differences between the leading and lagging double strands. As suggested in Figure 10b, arms with nicked lagging strands will have a random coil structure because the ~20 Okazaki fragments first have to become ligated (see Appendix C), whereas the leading arms can directly become supercoiled and transcribed. It can be assumed that once the four nascent arms in the initial replication bubble have formed separate blobs, their physical entanglement becomes unlikely because the mixing of such blobs is energetically unfavorable. Their de-mixed state in the parental DNA network could represent a minimal energy situation, just as the phase separation between DNA and the cytoplasm, described in Section 2.2, represents a minimal energy situation. Future investigations by polymer physicists should reveal whether such a de-mixed state between newly synthesized and parental DNA is feasible (see discussion in [83] and supplementary information in [36]).

It is proposed that the four nascent replichore arms continue to remain separated and form separate microdomains (Figure 10c) that enlarge and displace each other. This exclusion of chromosome arms may be helped by the low DNA diffusion coefficient, as determined in both liberated nucleoids [34] and living cells [84]. Because the mixing or entanglement of the newly synthesized replichores is prevented from the beginning, the process of entropic de-mixing [82] at a later stage may not be necessary.

An immediate separation of the origins in the replication bubble is to be expected if the replicated DNA in the origin region increases in mass and is free to move, while the two replisomes remain tethered to the unreplicated parental DNA they are reeling in. Such tethering of the replisomes would force the duplicated origins to move apart, as indicated by the double black arrow in Figure 10b. It can be envisaged that once the initial blobs have been established, they develop into larger separate domains that are fed by de novo DNA synthesis (Figure 10c). This causes the disappearance of the initial replication bubble, as visualized in Figure 10b, which may represent a structure with replisomes that differ from the ongoing ones (Figure 10c). Intriguingly, Khodursky et al. [85] may have referred to such a mechanistic distinction in their discussion of the effects of inhibiting the initiation of replication forks on thymineless death (TLD).

While the four microdomains enlarge through de novo DNA synthesis within the meshwork of unreplicated parental DNA, they are envisaged to first form a tetrahedron in the replication bubble (Figure 10c and Figure 11a). Further enlargement of the domains by the two replisomes will force the domains to displace each other and rearrange in the long axis of the narrow nucleoid (see Appendix C). Such a rearrangement will cause different patterns in cells with six spots, as depicted in Figure 9c. The domain rearrangement may be related to large-scale structural changes and rapid movement of certain loci (“snaps”), as described by Joshi et al. [86]. As previously proposed by Sherratt and coworkers [69], transient pausing of one replisome, causing a lower velocity of DNA synthesis of one replisome and thus, of domain expansion, may cause loci in the smaller domains to be pushed to mid-cell by the faster-expanding domains synthesized by the other replisome. This results in the patterns like R-O-L L-O-R or L-O-R R-O-L, as indicated in Figure 11(b2).

The transition to a faster growth rate (nutritional shift-up) is illustrated in Figure 11c. Because the cells become wider [87], there is no need for the enlarging microdomains to rearrange and adopt different side-by-side patterns. Instead, the tetrahedral conformation of microdomains is maintained, as previously described for spherical thymine-limited cells by Zaritsky et al. [88]. In addition, because no rearrangement of domains is necessary in the wider cells, there is no need for the origins to pass the replicating chromosome arms (see Figure 9b). As a result, the origins keep moving apart at the tip of the developing nucleoid toward the cell poles, even upon reinitiation, resulting in a longitudinal arrangement of chromosome arms, as described in [76].

Could this proposal for the formation of four excluding and expanding domains starting in the initial replication bubble (Figure 10a) be confirmed by the chromosome conformation capture technique or Hi-C [10], for instance, by increased interactions within the four domains? This would not be expected if the contacts occur as intra-arm interactions on the same replichore, visualized as the primary diagonal on the Hi-C contact map (for *E. coli*, see Figure 1A in [55]). If the domains of nascent right and left replichore arms were to intermingle, inter-arm interactions would be visualized as a secondary diagonal on the Hi-C contact map (for *C. crescentus*, see Figure 3Ad in [80]). The absence of such interactions on published *E. coli* Hi-C maps is in agreement with the four-excluding-arms model proposed here.

The organization of the chromosome in the four-excluding-arms model is compatible with the higher-order level of organization of the chromosome into four macrodomains (Ter, Ori, Right, and Left) and two nonstructured regions, as described in [89] (see also [55]). As soon as the left and right replichores have replicated and the four excluding microdomains (Figure 10b) have developed into four macrodomains, they will develop in the duplicated structured and unstructured macrodomains as described in [89] (see also [10]).

For the transversal arrangement of the chromosome, as depicted in Figure 11a, the asymmetric deposition of newly synthesized DNA requires stretched regions of replicated DNA that feed the newly developing nucleoids (see Figure 4B, panels 3 and 4 in [66]). These “feeding strands” can be expected to induce superdiffusive motion of loci, which has occasionally been observed [84,90].

**Figure A2:**
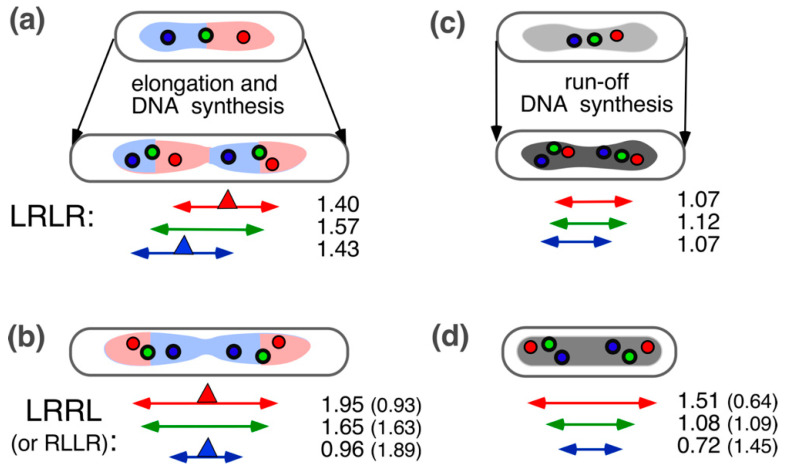
Graphical overview of the average distances between spot pairs (L-L, O-O, R-R) measured in growing and non-growing rifampicin-inhibited cells of *E. coli* K-12 (MG1655), as documented in Table 3 in [66]. The lengths of cells and segregation distances (colored double arrows) are drawn to scale. The numbers next to the double arrows indicate distances in µm, averaged over the first 5 constructs in Table 3 in [66]. Because the nucleoids were not visualized by staining, their sizes are not known and are drawn schematically. (**a**) In growing cells, showing the L-R-L-R ordering pattern of chromosome arms, replicated loci of the L-O-R pattern segregate over similar distances maintaining the L-O-R-L-O-R pattern (see Figure 9 in the main text). The red and blue triangles indicate hypothetical replisomes that have slightly moved apart. (**b**) In cells showing the LRRL or RLLR ordering pattern, the hypothetical replisomes stay in the cell center, and the L-L and R-R distances are either larger or smaller than the segregation distance between the origins (green O-O). (**c**,**d**) In rifampicin-treated cells, the loci segregate during run-off DNA synthesis similar to control cells (cf. **a**,**b**). Smaller cells have an elongated nucleoid that contains double the amount of DNA due to run-off DNA synthesis (indicated by the darker shade; see Figure 3 in [66]). Such nucleoids will not divide. Cell lengths and segregation distances are smaller than in growing cells (**a**,**b**).

Can the four-excluding-arms model explain the distances between replicated spots, as documented for the slow-growing *E. coli* K-12 cells in [66]? In Appendix C, the volumes of the developing domains are calculated for the slow-growing *E. coli* cells depicted in Figure 11a,b. Although the calculations apply to a different strain (*E. coli* B/r), the results show that the distances obtained by mere de novo DNA synthesis (1.8 µm in Figure A1, cell b-4) are similar to those measured in *E. coli* K-12 in [66] (1.4–1.6 µm in Figure A2a,b).

### 4.3. Comparison between Bacteria and Eukaryotic Cells and the Phenomenon of Cohesion

A fundamental difference between bacteria and eukaryotes is the pairing and alignment of sister chromatids throughout the eukaryotic G2 phase until the chromatids become condensed in the metaphase of mitosis [94]. During this cohesion period, the eukaryotic sister chromatids are held together by ring-shaped cohesin protein complexes, thus overcoming the gap between the time when the genome is replicated and when the chromatids are physically separated. However, during the S phase, sister chromatids become segregated through the loop extrusion activity of condensin II complexes, which prepares the chromosome for further condensation by condensin I [95]. Subsequently, the chromatids become separated by the proteolysis of cohesins and are transported by microtubules of the mitotic spindle, processes that do not occur in bacteria. Hirano [96] emphasized that “sister chromatids in eukaryotes are already well resolved by metaphase before they are subjected to poleward movement in anaphase” and that it is, therefore, reasonable to hypothesize that the resolution process in eukaryotes is mechanistically equivalent to the segregation process in bacteria (see also [83]).

Contrary to the proposition of Bates and Kleckner [42], it has been argued that there is no trustworthy experimental evidence for a eukaryotic cohesion phenomenon in bacteria [13,97] and that replicated DNA strands can immediately be separated if no precatenanes have been formed behind the forks that cause interstrand entanglements [98]. The estimation of cohesion time is generally based on independent measurements of, for instance, the copy number of the *gln* locus in comparison with its appearance as fluorescent foci in the cells [86] (for discussions of cohesion experiments in *E. coli*, see [99,100]).

While there seems to be no functional necessity for the initial linking of bacterial daughter strands by cohesion, analogous to cohesion in eukaryotes, the passive four-excluding-arms model presented here suggests an immediate separation of nascent chromosome arms in the replication bubble. However, an active mechanism for transiently bringing arms together at some distance from the replisome for processes such as recombination and mismatch repair can be imagined. Such an organization, involving SeqA complexes at a distance of ~250 nm from the replisome (cf. distances in Figure A1 and Table A3), has been described by Skarstad’s group [101].

## 5. Conclusions

An important point discussed in this review is the depletion of soluble proteins from the bacterial nucleoid as a result of the various macromolecular interactions between supercoiled DNA, soluble proteins, and polyribosomes. However, the polymer physics concepts and equations describing these interactions are difficult and not very accessible. The question of how to explain the low RI value observed by phase-contrast microscopy of *E. coli* cells (Figure 1, Figure 2 and Figure 3) was discussed in two studies [5,6], with different polymer physics starting points and different results concerning the role of depletion of soluble proteins from the nucleoid. A final conclusion has to await better agreement on the biological input values of the number of soluble proteins in slow-growing *E. coli* cells and better estimates of the volume of the nucleoid (see Figure 5). Regarding the polymer physics approaches to minimizing the total free energy of cells, it would be helpful if they were accompanied by a more informative explanation (see [35,36]) in order to understand the sources of the input values and the complicated computations.

Although the two studies [5,6] predict different behaviors of the nucleoid upon transcription inhibition by rifampicin, the microscopy studies summarized in Table 2 are inconclusive. Better microscopy of living cells is required to evaluate and understand the shape change in the nucleoid in rifampicin-inhibited cells.

Studies on bacterial DNA segregation indicate two different views; the resolution and movement of replicated chromosome arms occur either by a dedicated active process based on DNA loop extrusions through SMC complexes or by a passive process of de novo DNA synthesis, as described here using the four-excluding-arms model (Figure 10b,c). If the intermingling of newly synthesized DNA strands occurs in the initial replication bubble, it would be expected that entanglement could only be resolved with an elaborate mechanism involving topoisomerases and SMC proteins [20]. However, the different physical properties of nascent leading and lagging chromosome arms (Figure 10a), together with different gene expression activities between the two replichores, could prevent the mixing of the four replicated chromosome arms from the beginning. In that case, the key to segregation lies in the build-up of the replication bubble: if no initial mixing occurs due to their different physical properties, the four chromosome arms will exclude each other and become confined in four individual domains (Figure 10b) without the need for de-mixing. The role of topoisomerases and SMC proteins could be attributed to repair and recombination processes operating at some distance behind the replication forks [101] (see discussion in [15]).

The results of Hi-C interaction analyses [11,55] seem to confirm the four-excluding-arms model because interchain interactions between arms are negligible. However, so far, microscopic observations have not provided any indication of the existence of four excluding domains. Further development of techniques for pulse labeling of nascent DNA, as performed by Spahn et al. [51], seems promising if they can be applied to slow-growing *E. coli* cells. As described on Small Things Considered (https://schaechter.asmblog.org/schaechter/2019/11/forbearance-with-the-escherichia-coli-nucleoid.html; accessed on 14 March 2020), the Dekker group studied the dynamics of a ring-shaped nucleoid that had “opened up” and was replicating in artificially widened cells. The studies showed how, in living cells, duplicated origin spots are often positioned in or near low-density blob-like DNA domains [102]. In another study by this group [103], they showed how, upon initiation of DNA replication, the duplicated origins first move apart in random directions but then reorient toward the long axis after some time when the amount of replicated DNA has increased locally. These observations also indicate what might happen during the hypothetical transition from slow to fast growth, as depicted in Figure 11c,d.

Further development in spatial light interference microscopy [104] or digital holographic microscopy combined with optical diffraction tomography [27], as well as improved labeling techniques for nascent DNA strands [51], will be necessary to evaluate the hypothesis of the four excluding arms created in the initial replication bubble. When a more detailed quantification of the number of proteins involved in the replication bubble becomes available, calculations of the free-energy state of the proposed four nascent arms for the construction of the DNA replication cycle during shift-up, as performed by Odijk for the whole nucleoid (Figure 5 in [47]), could become possible. Such calculations might support the proposal of passive DNA segregation in terms of the hypothetical four excluding domains (Figure 10b) gradually replacing the parental nucleoid.

## Figures and Tables

**Table 2:** Microscopic studies from the literature of the effect of rifampicin on the spatial organization of the nucleoid in *E. coli* cells.

First Author	Cells and Growth Conditions (Growth Rate: Fast/Slow)	Rifampicin Treatment and Imaging/Preparation/ Staining	Appearance of Nucleoid or Interpretation (Figure in Reference)	Fully Dispersed Nucleoid ^(1)^ (Yes/No)
Dworsky [59]	*E. coli* K-12; M9 + glucose + Casa, 37 °C (fast)	100 µg/mL, 30 min, electron microscopy, and OsO_4_ fixation	Axial appearance (Figure 1b)	no
Harrington [60]	*E. coli* K-12 (NT3) LB medium, 37 °C (fast)	20 µg/mL, 60 min, light microscopy, poly-lysine slide, and no fixation	Nucleoids decondense (Figure 2D)	yes
van Helvoort [61]	*E. coli* K-12 (MC4100) Glucose minimal medium, 30 °C (slow)	100 µg/mL, 30 min, light microscopy, OsO_4_ fixation, and DAPI staining	Nucleoid fusion (Figure 4B)	no
Zimmerman [33]	*E. coli* K-12 (C600) LB medium, 37 °C (fast)	40 µg/mL, 60 min, and no fixation	Compact nucleoids (Figure 6B)	no
Cabrera [57]	*E. coli* K-12 (DJ2599) M63 + glucose + Casa, 30 °C (fast)	50 µg/mL, 10 min, DIC microscopy, formaldehyde fixation, and DAPI staining	Less condensed (Figure 2B)	yes
Sun [62]	*E. coli* K-12 M9 + glucose + Casa, 30 °C (fast)	100 µg/mL, 30 min, phase-contrast, methanol-fixed cells, and DAPI staining	Staining uniform throughout cells (Figure 3F)	yes
Cabrera [57]	*E. coli* K-12 (DJ2599) LB medium, 32 °C (fast)	100 µg/mL, 20 min, light microscopy, formaldehyde fixation, and DAPI staining	Quick expansion, elongated nucleoid, and phase-separated (Figure 1B)	no
Bakshi [63]	*E. coli* K-12 (MG1655) Low-phosphate EZRDM 30 °C Td = 60 min (slow)	200 µg/mL, 30 min, widefield epifluorescence microscopy, no fixation, and DNA stain DRAQ5	Radial compaction and axial expansion (Figure 7B)	no
Jin [54]	*E. coli* K-12 () LB medium, 37 °C (fast)	50 µg/mL, 30 or 60 min, and fixation.	Fully expanded nucleoid and phase separation (Figures 11 and 13)	no
Bakshi [64]	*E. coli* K-12 (MG1655) Low-phosphate EZRDM, 30 °C Td = 60 min (slow)	300 µg/mL, 20 min, phase-contrast microscopy, no fixation, time-lapse, and SYTOX orange staining	Radial contraction and axial contraction, followed by expansion (Figure 2)	no
Bakshi [65]	idem	idem	(Figure 8b)	no
Stracy [53]	*E. coli* MG1655 LB medium, 37 °C (fast)	50 µg/mL, 30 min, SI microscopy, no fixation, and DAPI staining	Nucleoid expansion (Figure 4C)	yes
Woldringh [66]	*E. coli* MG1655 (FH4035) Minimal glycerol medium (slow)	300 µg/mL, 210 min, 28 °C, fluorescence microscopy, OsO4 fixation, and DAPI staining	Compact nucleoids; 9% divided nucleoids (Figure 3B)	no
Spahn [51]	*E. coli* K-12 (MG1655/KF26 cells) LB medium, 32 °C (fast)	100 µg/mL (Sigma), 30 min, and fixation with 2% formaldehyde + 0.05% glutaraldehyde PAINT-SMLM imaging	Nucleoid contraction and expansion Nucleoid fusion? (Figure 5b)	no
Yang [7]	*E. coli* K-12 (MG1655) Slow: M9 glycerol minimal medium, Td = 60 min at 28 °C Moderately fast: M9 + glucose +Casa, Td = 30 min at 28 °C	300 µg/mL, 20–90 min, epifluoresc. microscopy, and no fixation	Length expansion at moderately fast growth (Figure 4) Volume expansion at fast growth (Figure S13)	Slow: no fast: yes
Xiang [9]	*E. coli* K-12 (MG1655) M9-glycerol + Casa, 37 °C (fast)	300 µg/mL, 40 min, no fixation, and DAPI staining	Nucleoid expansion and nucleoid fusion (Figure 7A)	yes
Chang [6]	*E. coli* K-12 (MG1655-JM57) EZ-Rich medium + glucose, 37 °C (fast)	300 µg/mL, 20–90 min, epifluoresc. microscopy, no fixation, and HupA-mNeon Green	Nucleoid expansion (Figure S4a)	yes
Spahn [58]	*E. coli* K-12 (NO34) LB at 32 °C (fast)	100 µg/mL, 60 min, CSL microscopy, and fixation with 2% formaldehyde + 0.05% glutaraldehyde	Contraction followed by expansion after 20 min to elongated structure (Figure 2B)	no

^(1)^ If “yes”, the DNA stain is dispersed throughout the whole cell, i.e., also in end caps. There is no visible phase separation between nucleoid and cytoplasm. If “no”, The nucleoid is still visible as a phase-separated structure, although its volume may have increased by run-out DNA synthesis or nucleoid fusion; its shape may have changed by expansion along the long axis (“axial filament”) and nucleoid contraction along the short axis (see explanation in [56]).

## Data Availability

Not applicable.

## Appendix A

1. Computation of theoretical values for protein volume fractions in nucleoid and cytoplasm and volume of the nucleoid

An *E. coli* cell is considered to be a compartment filled with a mixture of (i) a chromosome consisting of a single branched plectonemic DNA supercoil and (ii) small globular proteins. This mixed state is maintained at the cost of energy. Therefore, de-mixing or phase separation between proteins and DNA is to be expected, resulting in a minimal energy state at thermodynamic equilibrium. This implies a reduction in free energy in spite of the fact that the formation of a DNA phase (or nucleoid) appears to be more ordered.

To obtain values for the protein volume fractions in the nucleoid and cytoplasm and for nucleoid volume, Odijk [5] formulated equations for the free energy of the principal interactions between the superhelical segments of the DNA ($F_{self}$) and between the DNA double helix and soluble proteins ($F_{cross}$). Here, we follow the work of Theo Odijk [5], as also explained in [35,105]. The computation is performed in four steps:

(i) Define the excluded volumes of DNA supercoil segments colliding with themselves (Figure 4b in the main text) and DNA–protein cross-interactions (Figure 4c in the main text).
(ii) Take the derivatives of the free-energy equations with respect to the volume and number of proteins of the two phases. Equations of the force of compaction (osmotic pressure) and particle mixing (chemical potential) are obtained in both phases in the cytoplasm and nucleoid.
(iii) Equalize both forces for the two compartments (Figure 4d in the main text), resulting in two coexistence equations that contain the three unknown variables of protein volume fractions in the cytoplasm (*v_cyto_*) and nucleoid (*v_nuc_*) and the volume of the nucleoid (*V_nuc_*).
(iv) Together with a third equation on the volume fraction of total protein, compute the three variables and compare them to experimental values.

2. Excluded volume of DNA supercoil self-interactions

Here, we consider the superhelical DNA in the cell as it interacts with itself. As given in Table A1, the DNA supercoil has a diameter (*D_s_*) of 22 nm and a contour length (*L_s_*) of 640 µm [106]. The supercoiled DNA can be considered to consist of Kuhn segments with the step length or Kuhn length *A_s_ =* 158 nm (2× supercoil persistence length *P_s_*). These superhelical Kuhn segments collide with themselves, giving a strong excluded volume effect (blue and red supercoils in Figure 4b in the main text). The excluded volume (*b*) between two segments (see Figure 3 in [38]) can be estimated as follows (cf. Equation (2.5.2) in [105]):

(A1) $$b=2\;p\;{\left(\frac{1}{2}A_{s}\right)}^{2}\;D_{s}\;=\frac{1}{2}\;p\;{A_{s}}^{2}\;D_{s}$$

There are *N = Ls/As* Kuhn segments, giving rise to *N(N-1)/2* ≈ *N^2^/2* pairs of interactions. Therefore, the total excluded volume (*B_s_*) of the DNA supercoil (which is independent of *A_s_*) is as follows (cf. Equation (13) in [5]):

(A2) $$B_{s}\approx \left(\frac{\pi }{2}A_{s\;}^{2}D_{s}\right){\left(\frac{L_{s}}{A_{s}}\right)}^{2}/2\approx \frac{\pi }{4}L_{s}^{2}D_{s}$$

The superhelix self-energy, scaled by cell volume *V_cell_* and thermal energy *k_B_T,* can now be expressed as follows (cf. Equation (14) in [5]):

(A3) $${\;F}_{self}=\frac{\left(L_{s}^{2}D_{s}k_{B}T\right)}{Vcell}=\frac{B_{s}k_{B}T}{Vcell}$$

3. Excluded volume of DNA–protein cross-interactions

Next, we consider the statistical cross-interaction or steric repulsion between a spherical protein with an average radius (*a*) of 2.3 nm that is excluded from the DNA double helix (see Figure 3c in the main text). Because such a small particle will swim right through the interstitial space of the supercoil, the excluded volume must be proportional to the contour length *L* of the DNA double helix (*L* ≈ 1600 nm). Because of electrostatic repulsion, the protein cannot approach the double helix more closely than the depletion radius *E*, which can be estimated to represent the sum of the protein radius (*a*), the DNA-helix radius (1/2*d*), and two times the Debye screening length (*l*) for an ionic strength of 0.2 M:

(A4) $$E\approx a+\frac{1}{2}d+2\lambda \approx 2.3+1+1.36\approx 4.7\;nm$$

The protein exclusion volume, *B_cross_,* is the volume around the DNA cylinder, with radius *E* and length *L*, from which the protein is excluded or depleted (cf. Equation (8) in [5]):

(A5) $$B_{cross}\approx \pi E^{2}L$$

The total protein–DNA depletion energy of *m* proteins interacting with the DNA exclusion volume *B_cross_,* scaled by the cell volume *V_cell_* and thermal energy *k_B_T*, can be expressed as follows (cf. Equation (12) in [5]):

(A6) $$F_{cross}=\frac{mE^{2}Lk_{B}T}{Vcell}=\frac{mB_{c}k_{B}T}{Vcell}$$

4. Phase separation and coexistence equations

If the DNA is dispersed throughout the cell (volume *V*), the total free energy of the nucleoid, *F_nuc_*, is the sum of three energies:

*F_nuc_* = *F_mix_* + *F_cross_* + *F_self_*, (A7)

where *F_mix_* represents “an ideal mixing term due to the entropy of the proteins” (cf. Equation (5) in [34]). The above equation can be rewritten as follows (cf. Equation (15) in [5]):

(A8) $$\frac{F_{nuc}}{k_{B}T}=mln\frac{mv_{0}\;}{Vcell}-m+mg\left(\bar{v}\right)+\frac{{mB}_{c}}{Vcell\;}+\frac{B_{s}}{Vcell\;}\;$$

Here,$\;v_{0}$ is the volume of one protein with diameter *b*: $v_{0}=$ $\frac{4}{3}\pi {\left(\frac{1}{2}b\right)}^{3}=\pi b^{3}/6$ (*b* = 4.6 nm). Therefore, the volume fraction of the total protein ${v\;}_{tot}$= m.$v_{0}/Vcell$ or m/*V_cell_* = ${v\;}_{tot}$*/*$v_{0}$. Because the protein volume fraction in the cytoplasm (*m.*$v_{0}/V_{cell}$) is low, the interactions between proteins (*F_mix_*) are neglected. Also, the function *g(*$\bar{v})$ is not considered further. Here, it should be noted that *g (*$\bar{v})$ was not neglected in the calculations in [6].

Because *F_cross_* is ~10× *F_self_*, an unstable situation arises in the mixed suspension. By assuming a phase separation (cf. Figure 1 in the main text), a low-energy situation can be obtained in which the DNA occurs in a nucleoid with volume *V_nuc_*, together with soluble proteins. The general form of the rewritten equation (Equation (A8)) is now used to impose thermodynamic equilibrium between the cytoplasmic phase (with *m_cyto_* proteins and volume *V_cyto_*) and the nucleoid phase (with *m_nuc_* proteins and volume *V_nuc_*), where FcrossFself~ 1.

By taking the derivative of Equation (A8) with respect to volume, *V_cyto_*, we obtain the osmotic pressure (*π*) as a force for compaction. The derivative of energy with respect to the number of proteins, *m*, gives the chemical potential (*µ*) as a force for the mixing of molecules.

Rewriting Equation (A8) for the cytoplasm, we obtain the following:

(A9) $$\frac{F_{cyto}}{k_{B}T}=mln(\frac{m}{V_{c}})-m+m$$

Taking the derivative with respect to *m* gives the following:

(A10) $${\partial Fcyto/\partial m=\mu }_{c}=\ln\left(\frac{m}{V_{c}}\right)$$

Taking the derivative with respect to *V_cyto_* gives the following:

(A11) $$\partial Fcyto/\partial V=\pi_{c}=-\frac{m}{V_{c}}$$

Rewriting Equation (A8) for the nucleoid, we obtain the following:

(A12) $$\frac{F_{n}}{k_{B}T}=mln(\frac{m}{V_{n}})-m+mg+\frac{{mB}_{c}}{V_{n}}+\frac{B_{s}}{V_{n}}$$

Taking the derivative with respect to *m* gives the following:

(A13) $${\partial Fnuc/\partial m=\mu }_{n}=\ln\left(\frac{m}{V_{n}}\right)+\frac{B_{c}}{V_{n}}$$

Taking the derivative with respect to *V_nuc_* gives the following:

(A14) $${\partial Fnuc/\partial V=\pi }_{n}=\frac{m}{V_{n}}+\frac{m.B_{c}}{{V_{n}}^{2}}+\frac{B_{s}}{{V_{n}}^{2}}$$

To ensure a minimum energy situation, the chemical potential and osmotic pressure in the cytoplasm and nucleoid are equalized, giving two coexistence equations (cf. Equations (16) and (17) in [5]).

The two coexistence equations are obtained as follows:

For the equality of the osmotic pressure in the cytoplasm and nucleoid, we write π_c_ = π_n_ and obtain the following:

(A15) $$\frac{m}{V_{c}}=\frac{m}{V_{n}}+\frac{mB_{c}}{V_{n}^{2}}+\frac{B_{s}}{V_{n}^{2}}$$

Substituting $\frac{m}{V_{c}}$ with $\frac{v_{c}}{v0\;}$ and multiplying both sides of the equation by $\pi b^{3}/6$ gives the following (cf. Equation (16) in [5]):

(A16) $$v_{c}=v_{n}+\frac{B_{c}v_{n}}{V_{n}}+\frac{\pi b^{3}B_{s}}{6V_{n}^{2}}$$

For the equality of the chemical potential in the cytoplasm and nucleoid, we write *µ_c_* = *µ_n_* and obtain the following (cf. Equation (17) in [5]):

(A17) $$lnv_{c}=lnv_{n}+\frac{B_{c}}{V_{n}}\;$$

which is equivalent to: $ln\left(\frac{m}{V_{c}}\right)=ln\left(\frac{m}{V_{n}}\right)+\frac{B_{c}}{V_{n}}$.

In these equations, the relationship between the protein volume fraction (*v_c_* or *v_n_*) is used, as given by: $v=m\frac{prot.\;vol\;(51\;\times \;10^{-9})}{V}$ and the cytoplasmic or nucleoid volume (*V_c_* or *V_n_*).

In order to compute the unknown theoretical variables *v_c_* and *v_n_*, we solve Equation (A17) for *v_n_*. By eliminating *v_n_* from the two equations (substituting *v_n_* in Equation (A16) and dividing both sides by *v_c_*), they reduce to (cf. Equation (24) in [5]):

(A18) $$1-\frac{\pi b^{3}B_{s}}{6v_{c}V_{n}^{2}}=\left(1+\frac{B_{c}}{V_{n}}\right)e^{-B_{c}/V_{n}}$$

In order to compute the unknown theoretical variable *V_nuc_*, a third equation is needed, stating that the volume fraction of the total amount of protein (*v_tot_)* equals the sum of the protein volume fractions in the cytoplasm (*v_c_*) and nucleoid (*v_n_*):

(A19) $$v_{tot}V_{tot}=v_{c}{.V}_{c}+v_{n}.V_{n}=v_{c}\left(V_{tot}-V_{n}\right)+v_{n}V_{n\;}$$

The volume of the cell, *V_tot_*, is measured microscopically. Using the parameters given in Table A1, the theoretical values for protein volume fractions in the nucleoid (*v_nuc_*) and cytoplasm (*v_cyto_*) and for nucleoid volume (*V_nuc_*) can be computed. However, in the new calculations, the effect of (poly)ribosomes should be included, resulting in more complex equations; these will have to be published elsewhere.

From the protein and DNA concentrations in the two phases, the respective refractive indices (RIs) can be calculated, as shown in Appendix B.

**Table A1:** Input values for the computation of exclusion values *B_s_* and *B_c_* and free energies *F_self_* and *F_cross_*. For the number of soluble proteins (*m*) and volume of the average cell (*V_cell_)*, the values given in Figure 7b in main text were used.

Variable	Input Value
Volume of cell (cytoplasm + nucleoid), *V_cell_* (see Table 1, note (2) in the main text)	0.81 µm^3^
Double helix contour length, *L*	1600 µm
Superhelix contour length, *L_s_*	640 µm
Superhelix diameter, *D_s_*	22 nm
Average radius of 40 kDal protein, *a* Volume of spherical 40 kDal protein	2.3 nm 0.051 × 10^−6^ µm^3^
Supercoil Kuhn length, *A_s_*	158 nm
Number of supercoiled Kuhn segments, *N_s_ = L_s_/A_s_*	4000
DNA self-excluded volume *B_s_* = $\frac{\pi }{4}L_{s}^{2}D_{s}$	7073 µm^3^
*F_self_* = *B_s_*/*V_cell_* = 7073/0.81	0.87 × 10^4^ k_B_T
Number of soluble proteins, *m* (in the cytoplasm (0.77 × 10^6^) plus nucleoid (0.84 × 10^6^)) (see Figure 7b in the main text)	1.6 × 10^6^
Exclusion radius, *E (*$\approx a+\frac{1}{2}d+2\lambda \approx 2.3+1+1.36)$	≈4.7 nm
DNA–protein excluded volume, $B_{c}\approx \pi E^{2}L$ =	0.11 µm^3^
*F_cross_* = $\frac{mB_{c}}{Vcell}$	22 × 10^4^ k_B_T
Volume fraction total protein, $v_{tot}=m\frac{51\;\times \;10^{-9}}{V_{tot}}\;$	0.101

## Appendix B

1. Calculation of refractive index (RI) of cytoplasm and nucleoid from macromolecular concentrations

The refractive index is a (dimensionless) number (*n*) that indicates how the velocity of light (*v*) traversing an object with a certain density (*c*) is retarded with respect to its speed in vacuum. In the formula *n* = *c*/*v,* Barer and Joseph [23] established that the refractive index is linearly proportional to the concentration (density) of macromolecules in the cell. This is expressed by the formula *n_cell_ = n_water_ + g C*, where *C* is the macromolecular concentration (in grams/100 mL) and *g* is the specific refraction increment factor (in mL/g). More recently, it has been shown that this linear relationship between refractive index and concentration holds for complex cells [107] and bacteria [26].

To estimate the macromolecular concentration in the cytoplasm and nucleoid, we need to know (i) the volumes of the cell and nucleoid measured microscopically and (ii) the amounts of different macromolecules in the cell as determined by, among others, Churchward and Bremer [29]. Table A2 shows the theoretical refractive indices, calculated using the relationship described by Barer and Joseph [23].

In Table A2, the macromolecular composition and refractive indices of two slow-growing *E. coli* cells are compared: (i) *E. coli* K-12, as described by Männik’s group and discussed in Section 3.1, and (ii) *E. coli* B/r cells, as described in [5,28]. Schematic drawings of the two cell types are shown in Figure 7a,b, assuming the exclusion of polyribosomes from the nucleoid and no depletion of soluble proteins. The data are from DAPI-stained *E. coli* B/rH266 cells [40] (see last row of Table 1 in the main text), which were recently remeasured. Note that these measurements are different from those obtained by confocal scanning light microscopy, as published in [28] and described in Section 2.1 (see first row of Table 1 in the main text).

At present, there are two major uncertainties in the data we use: the volume of the nucleoid and the number of soluble non-ribosomal proteins. Assuming the exclusion of polyribosomes from the nucleoid without the depletion of soluble proteins by the nucleoid, the reduction in the RI value of the nucleoid compared to the cytoplasm (i) increases with increasing relative nucleoid volume and (ii) increases with decreasing concentration of soluble proteins.

**Table A2:** Estimation of macromolecular concentrations and refractive indices for two slow-growing *E. coli* populations, assuming equal dispersion of soluble proteins throughout the nucleoid and cytoplasm. Cell and nucleoid volumes and macromolecular concentrations are calculated for the average cell in the population (see Figure 7a,b in the main text).

Volume (× 10^−12 mL^), Mass (× 10^−12^ mg), and Numbers	*E. coli* K-12 [6] ^(1a)^	*E. coli* B/rH266 [40] ^(1b)^
Doubling time at 37 °C	60 min	150 min
Volume of the cell, *V_cell_*	0.7 ^(2a)^	0.81 ^(2b)^
Volume of the nucleoid, *V_nuc_*	0.35	0.33 ^(2b)^
Volume of the envelope, *V_env_*	-	0.12 ^(3b)^
Volume of cytosolic phase, *V_cyto_*	0.35 ^(4a)^	0.36 ^(4b)^
Volume of cytoplasm + nucleoid, *V_cyto+nuc_*	-	0.69 ^(5b)^
Total mass of proteins	22.59 ^(6a)^	136 ^(6b)^
Total mass of soluble and ribosomal proteins (considered as 40 kDa proteins)	15.3 ^(6a)^	116 ^(6b)^
Total number of 40 kDa proteins (soluble + ribosomal proteins)	0.34 × 10^6^	2.05 × 10^6^
Number of ribosomes	6000 ^(7a)^	8000 ^(7b)^
DNA mass (1 chromosome equivalent)	4.72 ^(8a,b)^	
Average number of chrom. equivalents per cell, *G_c_*	1.67	1.35 ^(9b)^
Total mass of stable RNA for 6000 ^(10a)^ or 8000 ^(10b)^ ribosomes	19.2 ^(10a)^	23 ^(10b)^
Mass of ribosomal proteins in the cytosol	7.32 ^(11a)^	9.76 ^(11b)^
Number of ribosomal proteins considered as 40 kDa proteins	0.11 × 10^6^	0.15 × 10^6^
Mass of non-ribosomal (soluble) 40 kDa proteins in the cytosol	8.1	106 ^(11b)^
Number of non-ribosomal (soluble) proteins in cytoplasm	$0.122\times 10^{6}$	0.84 × 10^6^
Number of non-ribosomal (soluble) proteins in nucleoid	0.108 × 10^6^	0.77 × 10^6^
Total number of non-ribosomal (soluble) proteins	0.23 × 10^6^	1.61 × 10^6^
**Concentrations (g/100 mL)** **^(12)^**		
DNA concentration in nucleoid (DNA mass × number chrom.equiv./*V_nuc_*)	2.25	1.93
Concentration of soluble proteins in nucleoid	2.05 ^(6a)^	15.4 ^(6b)^
Concentration of stable RNA in the cytosol, *V_cyto_*	5.49 ^(10a)^	6.39 ^(10b)^
Concentration of soluble proteins in the cytosol	2.32 ^(6a)^	15.4 ^(6b)^
Concentration of ribosomal proteins in the cytosol, *V_cyto_*	2.09 ^(11a)^	2.71 ^(11b)^

^(1a)^*E. coli* K-12 cells as described in [6] (see also [7]). ^(1b)^ *E. coli* B/rH266 cells [40] (see Figure 5 in the main text). Cells were stained with DAPI, prepared on an agar slab, and visualized by fluorescence microscopy. Previously [28], cells from the same strain were measured after attachment to a coverslip with poly-lysine and visualized with a CSLM. It appeared that the latter cells and nucleoids were significantly smaller for unexplained reasons (see Table 1 in the main text). ^(2a)^ See Table S1 in [6]. ^(2b)^ Average volumes of cells and nucleoids were obtained from a distribution of 281 cells. A threshold of 0.5 was applied using the ObjectJ plugin of Vischer: Coli-Inspector-04q+NucVol.ojj (see Figure 5b in the main text). ^(3b)^ Because the cell envelope is not accessible to diffusing proteins or particles, its volume was calculated assuming a total thickness of 23 nm (plasma membrane: 6 nm, peptidoglycan layer: 4 nm, and outer membrane: 13 nm). For an average cell, the volume *V_env_* = 0.12 × 10^−12^ mL, which is 15% of the cell volume *V_cell_.* ^(4a)^ The volume accessible to (poly)ribosomes, *V_cyto_,* is obtained from *V_cell_* − *V_nuc_.* ^(4b)^ *V_cyto_* obtained from *V_cell_* − *(V_env_ + V_nuc_).* ^(5b)^ The volume accessible to soluble proteins, *V_cyto+nuc_,* is obtained from *V_cell_* − *V_env_.* ^(6a)^ Based on the results of free energy minimization [6], the total number of soluble (non-ribosomal) 40 kDa proteins = 2.3 × 10^5^. Mass of 40 kDa protein = 40,000 × 1.66 × 10^−21^ = 66.4 × 10^−18^ mg. Total mass of (non-ribosomal) 40 kDa proteins = 2.3 × 10^5^ × 66.4 × 10^−18^ = 15.3 × 10^−12^ mg. Together with the ribosomal mass (note 11a) of 7.32 × 10^−12^, this gives a total protein mass of 22.59 × 10^−12^ mg (compared to 6b below). The mass of soluble non-ribosomal proteins is divided over the nucleoid with 1.08 × 10^5^ proteins (mass = 7.2 × 10^−12^ mg) and the cytosol with (2.3 − 1.08 =) 1.22 × 10^5^ proteins (mass = 8.1 × 10^−12^ mg). Concentration of 40 kDa proteins in nucleoid = 7.2 × 10^−12^/0.35 × 10^−12^ =20.5 mg/mL (2.05 g/100 mL). Concentration of 40 kDa proteins in cytosol = 8.1 × 10^−12^/0.35 × 10^−12^ = 23.14 mg/mL (2.32 g/100 mL). ^(6b)^ Based on the total mass of protein in Table 2 (note *l*) in [46], *P_C_* = 136 × 10^−12^ mg (mass of 40 kDa protein = 40,000 × 1.66 x 10^−21^ = 66.4 × 10^−18^ mg). Total number of 40 kDa proteins = 1.36 × 10^−12^/66.4 × 10^−18^ = 2.05 × 10^6^. Subtraction of proteins in volume envelope: assuming equal distribution of 40 kDa proteins over the envelope and cytosol + nucleoid, the envelope (with volume V_env_ = 0.15 × V_cell_) contains 0.15 × 136.10^−12^ = 20.4 × 10^−12^ mg protein. This amount is subtracted from the total protein mass (136 × 10^−12^ mg) because the envelope proteins cannot interact with DNA. This leaves (136 − 20.4=) 116 × 10^−12^ mg protein in the cytosol + nucleoid, i.e., both ribosomal and soluble proteins. Overall concentration of 40 kDa proteins in V_cyto+nuc_ = 116 × 10^−12^mg/0,69 × 10^−12mL^ = 168 mg/mL (including the mass of ribosomal proteins; see note 11b). ^(7a)^ Based on the best-fit values for Equation (2) [6] and Ehrenberg et al. [108] and Milo [109] (see Figure 1c in [6]). ^(7b)^ Based on Table 3 (note *p*) in [46]; see note 6b above. ^(8a,b)^ Excluding water molecules removed during polymerization; mass of 1 bp DNA = 618 Da. DNA mass 1 chromosome equivalent = 4.6 × 10^6^ bp × 618 × 1.66 × 10^−24^ = 4.72 × 10^−12^ mg. The value given in [43] is used throughout: 4.8 × 10^−12^ mg. ^(9b)^ *G_C_* was calculated using the equation in [44]: *Gc = Td/Cln2 (2^(C+D/Td^ – 2^D/Td^)*, with *Td* = 150’, *B* = 50’, C = 80’, and *D* = 30’. ^(10a)^ Total mass of stable RNA = 16 × 10^−15^ g for 5000 ribosomes. For 6000 ribosomes, mass = 19.2 × 10^−15^ g. With *V_cyto_* = 0.35 × 10^−12 mL^, this gives a concentration of RNA in the cytosol of 19.2 × 10^−12^/0.35 × 10^−12^ = 54.9 mg/mL (5.49 g/100 mL). ^(10b)^ Total mass of stable RNA per cell for 8000 ribosomes given in Tables 2 and 3 in [108] = 23 × 10^−12^ mg. Concentration of RNA in cytosol = 23 × 10^−12^/0.36 × 10^−12^ = 63.9 mg/mL (6.39 g/100 mL). ^(11a)^ Mass of ribosomal proteins in the cytosol for 6000 ribosomes according to Ortega [110] = 6000 × (0.45 + 0.77) × 10^−15^ = 7.32 × 10^−12^ mg. This gives a concentration of 7.32 × 10^−12^/0.35 × 10^−12^ = 20.91 mg/mL (2.09 g/100 mL) in the cytosol. ^(11b)^ Mass of ribosomal proteins in the cytosol for 8000 ribosomes = 9.76 × 10^−12^ mg. This gives a concentration of ribosomal proteins in the cytosol of 9.76 × 10^−12^/0.36 × 10^−12^ = 27.1 mg/mL (2.71 g/100 mL). Mass of non-ribosomal soluble proteins = mass of 40 kDa proteins (see note 6b) - mass of ribosomal proteins (see above) = 116 × 10^−12^ − 9.76 × 10^−12^ = 106 × 10^−12^ mg. Assuming equal dispersion of soluble proteins through the cytoplasm and nucleoid, their concentration in *V_cyto+nc_* = 106.2 × 10^−12^/0.69 × 10^−12^ = 154 mg/mL (15.4 g/100 mL). ^(12)^ Theoretical refractive indices were calculated according to *n_cell_ = n_water_ + g r*, where *r* is the dry weight content per cell or cytoplasmic or nucleoid volume (expressed as g/100 mL) and *γ* is the specific refraction increment factor given by Barer and Joseph [23]. The same formula given in [28] was used: *n_cell_ = n_water_* + 0.0016 × nucleic acid (g/100 mL) + 0.00186 × protein (g/100 mL) + 0.00178 × remaining low-molecular-weight (LMW) compounds (5.8 g/100 mL).

The calculations of the refractive index (*n*) below indicate that for the *E. coli* K-12 cells of Chang et al. [6], the exclusion of polyribosomes and equal concentrations of soluble proteins in the nucleoid and cytosol (see Figure 7a) result in a reduction of the refractive index as contributed by a macromolecular content of 33%. This is in accordance with the observations in [28] (see last rows of Table A2 for the various concentrations used):

*n_nuc_* = 1.333 + 0.0016 × 2.25 (DNA) + 0.00186 × 2.05 (prot.) + 0.00178 × 5.8 (LMW) = 1.351

*n_cyto_* = 1.333 + 0.0016 × 5.49 (RNA) + 0.00186 × (2.32 + 2.09) (prot.) × + 0.00178 × 5.8 = 1.360

Reduction (*n*) as contributed by the macromolecular content: *n − 1.333* = (0.027 − 0.018)/0.027 = 0.33.

For the *E. coli* B/rH266 cells [40], the exclusion of polyribosomes and equal concentrations of soluble proteins in the nucleoid and cytosol (see Figure 7b) result in a reduction of only 22%:

*n_nuc_* = 1.333 + 0.0016 × 1.93 (DNA) + 0.00186 × 15.4 (prot) + 0.00178 + 5.8 (LMW) = 1.375

*n_cyto_* = 1.333 + 0.0016 × 6.39 (RNA) + 0.00186 × 15.4 + 2.71 (prot.) + 0.00178 × 5.8 =1.387

Reduction (*n*) as contributed by the macromolecular content: *n − 1.333* = (0.054 − 0.042)/0.054 = 0.22.

To obtain a difference of about 30%, as observed in [28], additionally depleting soluble proteins from the nucleoid to a concentration of ~13 mg/mL and increasing the protein concentration (soluble and ribosomal) in the cytoplasm to ~20 mg/mL are assumed to obtain a reduction of about 34% in RI in the nucleoid:

*n_nuc_* = 1.333 + 0.0016 × 1.93 (DNA) + 0.00186 × 13.4 (prot) + 0.00178 + 5.8 (LMW) = 1.375

*n_cyto_* = 1.333 + 0.0016 × 6.39 (RNA) + 0.00186 × 17.4 + 2.71 (prot.) + 0.00178 × 5.8 =1.387

Reduction (*n*) as contributed by the macromolecular content: *n − 1.333* = (0.058 *−* 0.038)/0.058 = 0.34.

## Appendix C

1. Calculation of domain volumes for the four-excluding-arms model

The starting point of the volume calculations of domains includes (i) the volume of the whole chromosome (contour length *L* = ~1600 µm) as estimated for a newborn *E. coli* B/r cell, described in Table 1 (last column) and Figure 5b in the main text: *V_nuc_* = 0.24 µm^3^, and (ii) the superhelix contour length of the non-replicating chromosome *L_s_* = 0.4 × 1600 = 640 µm, with a number of superhelical Kuhn segments of 4000 (see Table A1 in Appendix A). In addition, it is assumed that the speed of the *E. coli* Pol III replicase is about 1000 nucleotides (nt) per second [111] and that the length of Okazaki fragments is about 1500 nt [112]. Therefore, an average Okazaki fragment will be synthesized in 1.5 s. Thus, 30 s after initiation of DNA replication at *ori*, the lagging strands in the replication bubble will show a run of about 20 Okazaki fragments (Figure 10a in the main text). The ligation of Okazaki fragments involves removing RNA primers by endonucleases, filling the gaps between the fragments with repair DNA polymerase, and sealing them with DNA ligase. During this maturation process, nicked lagging strands cannot become supercoiled and will have the conformation of a random coil (Figure 10b in the main text); similar calculations were performed in [99].

After 30 s of DNA replication, 30,000 nt are synthesized by the leading and lagging strand Pol III replicases. Each newly synthesized strand has a length of 30,000 × 0.34 nm = ~10 µm of DNA (0.34 is the axial distance between base pairs). Assuming that this DNA becomes immediately supercoiled for the leading strand, it will represent a length of (0.4 × 10 =) 4 µm of supercoiled DNA. As the chromosomal contour length of supercoiled DNA represents 4000 Kuhn segments (Appendix A, Table A1), the 4 µm of supercoiled DNA synthesized in 30 s will contain about 25 Kuhn segments. These are depicted in Figure 10b of the main text as being folded into a blob, with a volume of 0.0015 µm^3^ and a diameter of 140 nm (see also Figure 4c in [8]), assuming that its packing density is the same as that of the whole nucleoid, which is 4000 Kuhn segments in a volume of 0.24 µm^3^.

After 5 min (300 s) of replication with a fork speed of 1000 nt/s, 300,000 × 0.34 nm = ~100 µm of DNA has been synthesized by each Pol III replicase of leading and lagging strands. This implies a contour length of supercoiled DNA for the leading strands of (0.4 × 100 =) 40 µm, folded into a microdomain of 250 Kuhn segments with a volume of 0.015 µm^3^ and a diameter of 310 nm (Figure 10c in the main text). See Table A3 for domain volumes obtained after longer replication periods.

**Table A3:** Domain volumes of the nucleoid after different periods of replication in slow-growing *E. coli* B/r cells (cf. Figure 11a,b in the main text).

Time after Initiation	Length of Newly Replicated DNA per Domain ^(1)^ (µm)	Approximate Volume of Nascent Domain ^(2)^ (µm^3^)	Domain Diameter of Sphere or Diameter/Length of Cylinder (µm)	Used for Panels in Figure A1b
30 s	10	0.0015	0.14	1
5 min	100	0.015	0.31	2
10 min	200	0.031	0.39	-
20 min	400	0.062	0.46/0.53	3
30 min	600	0.093	0.46/0.7	-
40 min	800	0.124	0.46/0.9	4

^(1)^ See Figure 10a,b in the main text. ^(2)^ Assuming the same packing density that holds for the chromosome (~1600 µm) in a nucleoid volume (0.24 µm^3^) for a newborn cell (see Table 1, last row in main text).

2. Segregation distances measured between replicated loci pairs in growing and non-growing cells

Distances in live unfixed *E. coli* cells constructed by Flemming Hansen as described in [66] were estimated. The four-excluding-arms model explains the observed patterns of replichores (Figure A1b) as well as the segregation distances measured between replicated loci pairs (Figure A1(b4)).
